# The role of the IL-9‒NLRP3 axis in insulin resistance and adipose tissue inflammation during diet-induced obesity

**DOI:** 10.1038/s41423-025-01340-4

**Published:** 2025-09-18

**Authors:** Marc P. Hübner, Dennis de Coninck, Benjamin Lenz, Jayagopi Surendar, Marianne Koschel, Narcisse Victor Tchamatchoua Gandjui, Beng Amuam Andrew, Lucy Cho Nchang, Anita Obi Bate Ebob, Fanny fri Fombad, Lisa Marie Springer, Lars Eppe, Frank A. Schildberg, Samuel Wanji, Achim Hoerauf, Alexander Pfeifer, Indulekha Karunakaran

**Affiliations:** 1 Institute for Medical Microbiology, Immunology and Parasitology, University Hospital Bonn, Bonn, Germany; 2 German Center for Infection Research (DZIF), partner site Bonn-Cologne, Bonn, Germany; 3 Institute of Pharmacology and Toxicology, University Hospital Bonn, Bonn, Germany; 4 Department of Orthopedics and Trauma Surgery, University Hospital Bonn, Bonn, Germany; 5 Parasite and Vector Biology Research Unit, Department of Microbiology and Parasitology, Faculty of Science, University of Buea, Buea, Cameroon; 6 Research Foundation in Tropical Diseases and the Environment, Buea, Cameroon; 7 University of Oslo, Oslo, Norway

**Keywords:** IL-9 signaling, Obesity, Inflammasome, Helminths, Inflammation, insulin resistance, Chronic inflammation, Predictive markers

## Abstract

Despite the proven beneficial role of type 2 cytokines in diabetes and obesity, IL-9, a predominant Th2 cytokine, has not been investigated in this context. The present study characterized the role of IL-9 signaling in obesity and metabolic dysfunction. We found decreased IL-9 levels in human type 2 diabetes patients and decreased IL-9 signaling in high-fat diet (HFD)-induced obese mice. On the other hand, recombinant IL-9 (rIL-9) treatment reversed insulin insensitivity and inflammation following HFD consumption. IL-9R knockout (KO) mice fed a HFD presented faster weight gain, impaired glucose and insulin tolerance, defective insulin signaling, increased adipocyte size, and decreased energy expenditure. In the adipose tissue of HFD-fed IL-9R KO mice, a significant increase in the number of CD11c+ macrophages and a decrease in the number of RELMα+ macrophages, eosinophils and ILC2s were observed, along with increased TNF, decreased adiponectin production and increased expression of NLRP3. In vitro treatment of human and mouse macrophages with rIL-9 decreased the release of NLRP3-induced IL-1β and IL-18. In vivo treatment of HFD-fed IL-9R KO mice with a pharmacological inhibitor of the NLRP3 inflammasome rescued body weight, insulin sensitivity and adipose tissue inflammation. Mechanistically, the STAT5 protein was found to be important for the IL-9-induced inhibition of the NLRP3 inflammasome in adipose tissue. In addition, we also demonstrated a potential role for IL-9 in the protective effects of helminth immunomodulation during obesity and insulin resistance in filaria-infected humans and in an animal model. Taken together, the results of this study highlight that IL-9 signaling improves insulin signaling by inhibiting NLRP3-induced inflammation.

## Introduction

The growing twin epidemics of obesity and type 2 diabetes represent some of the most significant global health challenges today [1]. The recognition of a heightened inflammatory state as a critical contributor to obesity and insulin resistance [2] has led to the reframing of obesity as an inflammatory condition. As a result, there has been a shift toward understanding the nature of this characteristic low-grade inflammation in the hope of modifying it to treat obesity and insulin resistance. Hence, cytokines of the interleukin family have emerged as one of the most important inflammatory mediators of obesity and metabolic health, with diverse and often opposing roles in either driving or inhibiting inflammation. While proinflammatory cytokines such as IL-1β are involved in the pathogenesis of obesity and insulin resistance [3], type 2-associated cytokines such as IL-13 [4], IL-4 [5], and IL-5 [6] are known to promote metabolic health. However, the impact of cytokines on obesity and insulin resistance development is much more complex than the pro- vs. anti-inflammatory balance; for example, the proinflammatory cytokine IL-36 was shown to alter the intestinal microbiome and to mediate protection against obesity and metabolic dysfunction [7]. Several clinical trials have demonstrated the consistent beneficial effects of anti-inflammatory agents such as an IL-1R antagonist (anakinra) and IL-1β-specific antibodies (gevokizumab and canakizumab) [8, 9] on HbA1c, insulin sensitivity and β-islet cell secretory function. Despite the proven protective roles of type 2 cytokines in obesity and insulin resistance, IL-9 signaling has never been investigated in this setting, except for one report showing reduced levels of IL-9 in type 2 diabetes patients [10]. However, IL-9, a classic type 2 cytokine, has been relatively well studied in the context of allergic immune responses, parasitic helminth infections and autoimmune diseases. It has been shown to induce host protection and central role against parasitic helminth infections by mediating mast cell proliferation and activation [11], isotype switching [12], promoting eosinophilia [13], epithelial cell hyperplasia [14] and worm expulsion [15]. Furthermore, with the identification of two new natural and exclusive IL-9 producers, a Th9 subset [16] and type 2 innate lymphoid cells (ILC2s) [17], source- and context-dependent variations in IL-9 function have been revealed. In addition, more complexity is added by the fact that IL-9 from ILCs can act differently in the induction and resolution phases of arthritis [18]. Likewise, in scenarios of autoimmunity, such as colitis [19], as well as in a mouse model of multiple sclerosis [20], IL-9 has also been demonstrated to play a proinflammatory role.

IL-9 has been shown to promote IL-5 [21] and IL-13 signaling [22], which have already been demonstrated to improve insulin sensitivity during diabetes. Moreover, an earlier report revealed reduced IL-9 levels in diabetes [10], and RNA sequencing data showed IL-9R expression in insulin target cells such as adipose tissue [23] and the liver [24]. We hypothesize that IL-9 plays a role in obesity-induced inflammation and thereby insulin resistance. In this study, we show that serum IL-9 levels are decreased in subjects with diabetes. Additionally, IL-9 correlated negatively with body fat and C-reactive protein (CRP) levels but correlated positively with insulin levels and muscle mass. These findings indicate that this cytokine plays a protective role in insulin resistance and obesity. These protective effects were also recapitulated in mice deficient in the IL-9 receptor (IL-9R KO), which presented increased weight gain, metabolic dysfunction, increased adipose tissue inflammation and decreased energy expenditure. Mechanistically, the ability of IL-9 signaling to inhibit the NLRP3 inflammasome in adipose tissue via the STAT1 and STAT5 proteins explains its protective effects in adipose tissue.

The hygiene hypothesis postulates that improved hygiene and subsequent loss of helminth infections and their immunomodulatory functions are attributed to the sharp increase in autoimmune diseases and allergies over recent decades. This logic can be extended to metabolic diseases such as type 2 diabetes, and several reports have shown a decreased incidence of diabetes in helminth-infected individuals [25–27]. Our group has previously shown that experimental helminth-induced immunomodulation can improve sepsis survival [28] and protect against atherosclerosis [29], type 1 diabetes [30–32] and diet-induced insulin resistance [33]. During diet-induced obesity, infection with the rodent filarial nematode *Litomosoides sigmodontis* and treatment with filarial antigens improved glucose and insulin tolerance and increased the immune cell populations associated with a lean, insulin-sensitive phenotype [33]. Recently, our group also showed that adipocyte-conditioned media from filarial antigen-treated mice modulates Th1 and Th17 frequencies in an adiponectin-dependent manner [34]. In human lymphatic filariasis, parasite antigen-specific Th9 cells have been shown to be associated with clinical pathology [35]. On the basis of the type 2-dependent helminth-mediated protection observed during diet-induced insulin resistance and the central role of IL-9 in driving host protective immunity against helminths, we hypothesized that helminths might act via IL-9 to induce insulin sensitivity during diet-induced insulin resistance. Our findings in an animal model as well as human data suggest that the protective effect of helminth infection on insulin resistance and obesity might be partly mediated by IL-9 signaling.

## Results

### Decreased serum IL-9 levels in type 2 diabetes patients

**Fig. 1:**
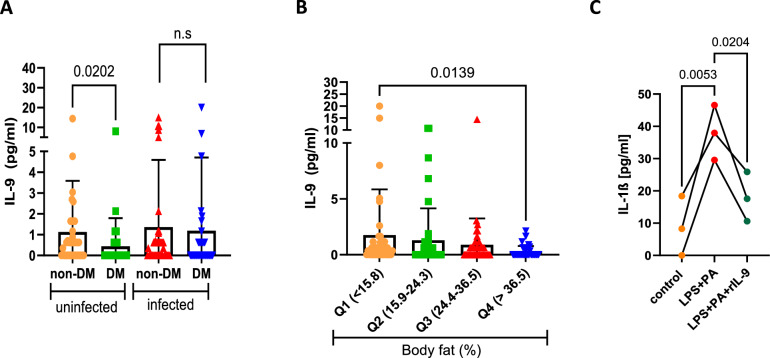
Decreased serum IL-9 levels in type 2 diabetes patients: **A** Serum levels of IL-9 in uninfected nondiabetes (non-DM, *n* = 38), uninfected diabetes (DM, *n* = 38), infected nondiabetes (non-DM, *n* = 47) and infected diabetes (DM, *n* = 36) participants; **B** IL-9 levels in quartiles of body fat in the total study cohort. **C** Human monocyte-derived macrophages from 3 donors were pretreated with human rIL-9 and treated with LPS for 3 h and palmitic acid for 18 h, and IL-1β levels were measured via ELISA. The data were analyzed via SPSS software, and the Kruskal‒Wallis test with Dunn’s multiple comparison test was used to compare the groups

To determine whether IL-9 is associated with insulin resistance and diabetes and to assess its role in helminth-induced immunomodulation during diabetes, we investigated the serum levels of IL-9 in a cohort of participants with or without diabetes who were infected with one of the following filarial nematodes: *M. perstans, O. volvulus* and/or *L. loa*. Among the infected participants, the group with diabetes had a significantly lower prevalence of *M. perstans* (24.4%; *p* < 0.001) and *L. loa* (28.2%, *p* = 0.006) in comparison to non-diabetic participants (Supplementary Table 1). However, both the nondiabetic and diabetic groups presented a similar prevalence of *O. volvulus*. Supplementary Table 1 shows the clinical and biochemical parameters of the study subjects. As expected, the diabetes cohort presented higher levels of HbA1c and increased CRP levels in both the infected and uninfected groups. The participants with diabetes were also slightly older and had higher CRP and triglyceride levels compared with the participants without diabetes. IL-9 levels were significantly lower in the uninfected diabetes subjects compared with their corresponding uninfected nondiabetic control group. However, the serum levels of IL-9 were comparable between subjects with and without diabetes in the infected group (Fig. 1A). These findings suggest that IL-9 might play a protective role during type 2 diabetes and that filarial infections might prevent the diabetes-associated reduction in IL-9 levels. Thus, IL-9 might be a factor responsible for the protective effect of helminth infection during diabetes. In line with this, the infected group with diabetes presented a slightly better metabolic profile, as shown by lower BMI, body fat, higher muscle mass, and lower HbA1c levels, compared with the uninfected diabetes participants (Supplementary Table 1). IL-9 levels also showed a significant but negative correlation with body fat and CRP levels and a positive correlation with muscle mass and insulin levels (*p* = 0.052), as shown in Supplementary Table 2. Specifically, among diabetic participants who were overweight or obese, IL-9 levels were negatively correlated with cholesterol levels. Since there are no population-specific cutoff points for body fat, we assessed IL-9 levels in increasing quartiles of body fat. With increasing quartiles of body fat, there was a significant decrease in the levels of IL-9 (Fig. 1B), again indicating that IL-9 might play a protective role during obesity. Similarly, in human monocyte-derived macrophage cultures, treatment with recombinant IL-9 (rIL-9) decreased the levels of IL-1β (Fig. 1C).

### Altered IL-9 signaling during obesity and rIL-9 treatment rescues insulin sensitivity and inflammation

**Fig. 2:**
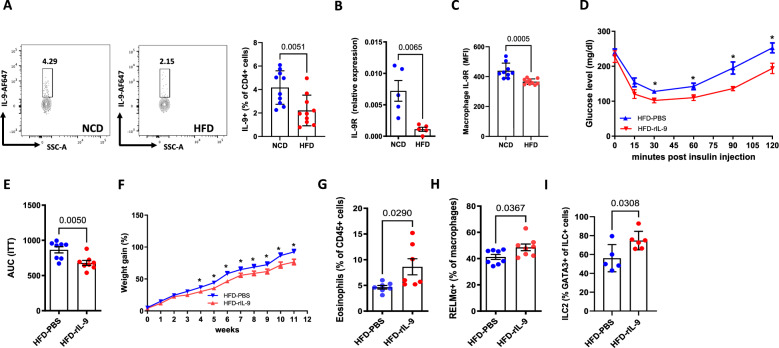
Altered IL-9 signaling during obesity and improved metabolic and inflammatory profiles in response to rIL-9 treatment: Mice were fed a normal chow diet (NCD) or high-fat diet (HFD) for 12 weeks, and **A** frequencies of IL-9 + CD4+ T cells in the SVF of adipose tissue were analyzed; **B** gene expression of IL-9R in the adipose tissue of NCD-fed and HFD-fed mice; **C** MFI of IL-9R in the adipose tissue macrophages of NCD-fed and HFD-fed mice; **D** insulin tolerance test (ITT) of HFD-fed mice treated i.p. with PBS or 2 µg/kg rIL-9 thrice weekly; **E** the area under the curve (AUC) of the ITT; **F** weight gain; **G** Adipose tissue eosinophils; **H** RELMα+ macrophages and **I** ILC2 frequencies in HFD-fed mice treated i.p. with PBS or 2 µg/kg recombinant IL-9 thrice weekly. All experiments were repeated twice, and representative data are shown. *p < 0.05, compared with HFD-fed PBS by two-way ANOVA. For **D** and **F**, two-way ANOVA was performed, and for the remaining two-tailed, unpaired t tests were performed

To investigate whether IL-9 plays a role in the development of obesity and insulin resistance, we investigated the frequencies of IL-9 in several immune cell types, such as B cells, eosinophils, CD4 + T cells, macrophages and ILC2s, in the adipose tissue of normal control diet (NCD) and high-fat diet (HFD) mice after 12 weeks of diet. We detected decreased frequencies of IL-9 + CD4+ cells (Fig. 2A) and IL-9 + ILC2s (Supplementary Fig. 1C) in adipose tissue. However, the frequencies of IL-9 in other cell types, such as B cells and macrophages, were comparable in the adipose tissue of the NCD-fed and HFD-fed mice (Supplementary Fig. 1D–E). Next, we probed IL-9R gene expression and detected decreased expression in the adipose tissue of HFD-fed mice (Fig. 2B). To identify the major source of IL-9R expression, we investigated CD3+ T cells, eosinophils, macrophages, NK cells, CD4+ T cells, ILC2s, and dendritic cells (Supplementary Fig. 1F). Among these cell types, IL-9R expression was highest in macrophages (Supplementary Fig. 1F), and during HFD, IL-9R expression was significantly decreased in macrophages (Fig. 2C). However, in all the other cell types studied, IL-9R expression was comparable between the NCD and HFD groups (Supplementary Fig. 1G–K). Taken together, during obesity, IL-9 production by CD4 + T cells and ILC2s and IL-9R expression on macrophages are significantly downregulated. Having observed decreased IL-9 levels during obesity, we next examined whether the administration of rIL-9 to HFD-fed WT mice might improve the hallmarks of insulin resistance, such as weight gain, impaired glucose tolerance and HFD-induced inflammation. While rIL-9 treatment had no significant effect on glucose tolerance (Supplementary Fig. 1A, B), a significant improvement in insulin tolerance, as assessed in vivo by the insulin tolerance test (ITT) (Fig. 2D, E), was observed. The body weight also decreased in the mice treated with rIL-9 (Fig. 2F). Furthermore, rIL-9 treatment increased the frequencies of eosinophils (Fig. 2G), RELMα+ macrophages (Fig. 2H) and ILC2s (Fig. 2I) in the adipose tissue, indicating that rIL-9 administration significantly improved parameters associated with diabetes in a model of HFD-induced insulin resistance.

### IL-9R KO mice have an impaired metabolic profile during steady-state and high-fat conditions

**Fig. 3:**
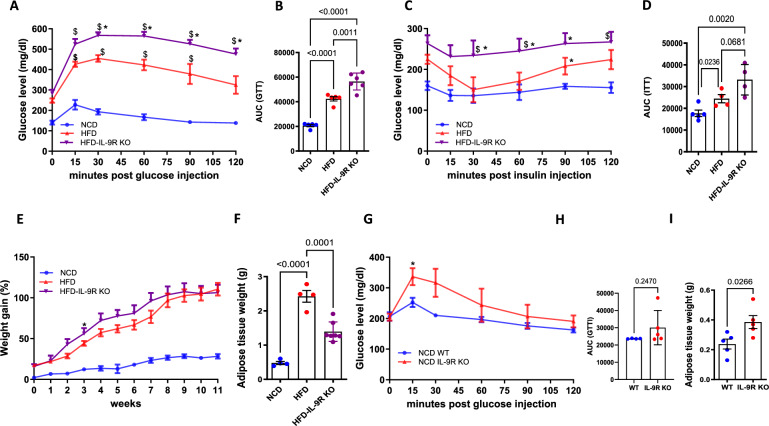
IL-9R deficiency leads to a worsened metabolic status during HFD consumption. **A** Glucose tolerance test (GTT) and **B** AUC for the GTT; **C** insulin tolerance test (ITT) and **D** AUC for the ITT; **E** body weight gain; **F** epididymal adipose tissue weight of mice fed a NCD, HFD or HFD-IL-9R KO for 12 weeks. **G** GTT; **H** AUC for GTT; **I** adipose tissue weights of WT and IL-9R KO mice fed a NCD for 12 weeks. All experiments were repeated twice, and representative data are shown. $ *p* < 0.05 compared with the NCD group; *p < 0.05 compared with the HFD  group, as determined by two-way ANOVA. For **A**, **C**, **E**, and **G**, two-way ANOVA was performed, and for the rest, one-way ANOVA was performed

To better understand how IL-9 might affect the pathogenesis of diet-induced obesity and insulin resistance, we used mice deficient in IL-9/IL-9R signaling. Wild-type (WT) and IL-9 receptor-deficient (IL-9R KO) mice were fed a HFD for 12 weeks, and littermates fed a normal chow diet served as lean controls. HFD-fed IL-9R KO mice presented significantly impaired glucose (Fig. 3A, B) and insulin tolerance (Fig. 3C, D) and faster weight gain but decreased adipose tissue weight (Fig. 3E, F) compared with HFD-fed WT controls. However, the size of adipocytes was increased in the HFD-fed IL-9R KO mice compared with WT control mice (Supplementary Fig. 2A, B), and the insulin-stimulated pAkt/Akt ratio was lower in the HFD-fed IL-9R KO mice than in the control mice (Supplementary Fig. 2C). In primary white adipocyte cultures treated with LPS and palmitic acid (PA), the most abundant fatty acid in the body, rIL-9 treatment rescued adiponectin secretion to control levels (Supplementary Fig. 2D). Furthermore, in primary white adipocyte cultures from IL-9R KO mice, the LPS + PA-induced TNF levels (Supplementary Fig. 2E) were significantly greater than those in the corresponding WT controls. However, there was no effect of rIL-9 treatment on the secretion of other cytokines and adipokines, such as leptin, IL-6, and IL-1β, in primary adipocytes (data not shown). The observations of decreased adipose tissue weight but increased dysfunction indicate ectopic fat accumulation, as suggested by increased systemic cholesterol (Supplementary Fig. 2F, *p* = 0.06) and hypertrophy of adipocytes owing to imbalanced hypertrophy and hyperplasia [36]. To explore whether the effect of IL-9R deficiency persists even under normal physiological conditions, we assessed weight gain and performed GTT and ITT in WT and IL-9R KO mice under normal chow conditions at 12–14 weeks of age, a time point at which the HFD-fed mice were analyzed. There was only a slight worsening of glucose tolerance (Fig. 3G, H) and insulin tolerance (Supplementary Fig. 2G, H) in the IL-9R KO mice and an increase in adipose tissue weight (Fig. 3I). There was a slight but non-significant increase in the number of total immune cells (Supplementary Fig. 2I) and a decrease in the eosinophil population in the adipose tissue (Supplementary Fig. 2J). These data suggest that the metabolic dysregulation and inflammation characteristic of IL-9R deficiency are already evident under steady-state conditions but become more pronounced upon exposure to a metabolic stressor such as a HFD.

### Decreased energy expenditure in the IL-9R KO mice

**Fig. 4:**
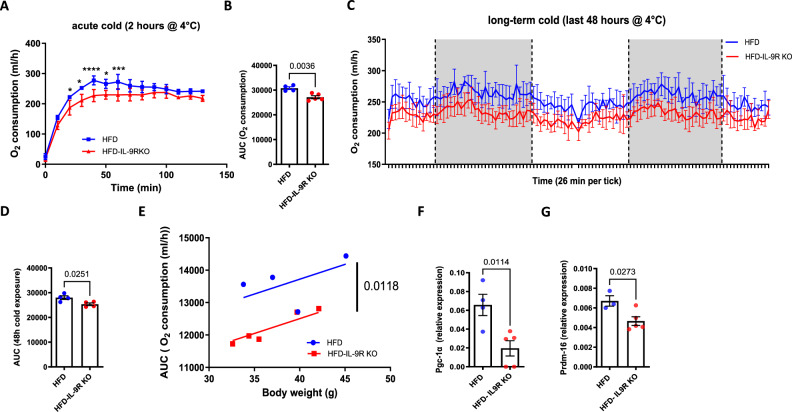
Decreased energy expenditure in mice with IL-9R deficiency. **A** O_2_ consumption during acute cold exposure (2 h at 4 °C) in obese wild-type (HFD) and IL-9R KO (HFD-IL-9R KO) mice; **B** area under the curve (AUC) analysis for **A**; **C** O_2_ consumption during the last 48 h of long-term cold exposure (3 days at 16 °C followed by 7 days at 4 °C) in the abovementioned groups; **D** AUC analysis for **C**; **E** relationship between body weight and oxygen consumption analyzed via ANCOVA of the abovementioned groups; gene expression of **F** Pgc-1α and **G** Prdm-16 in the brown adipose tissue of wild-type and IL-9R KO mice fed a HFD for 12 weeks after long-term cold exposure. All experiments were repeated twice, and representative data are shown. For **A**, **C**, two-way ANOVA was performed, and for **B**, **D**, **F**, and **G**, two-tailed unpaired *t* tests were performed

We next sought to determine whether the alterations in insulin sensitivity and inflammation are related to changes in energy expenditure. For this purpose, indirect calorimetry using metabolic cages was performed with HFD-fed IL-9R KO mice and age-matched controls. Oxygen consumption after acute cold exposure was significantly lower in the IL-9R KO mice than in the WT control mice (Fig. 4A, B). Moreover, after long-term exposure to cold, the IL-9R KO mice presented significantly lower oxygen consumption rates than the WT controls did (Fig. 4C–E). IL-9R KO mice also lost less body weight during long-term cold exposure (Supplementary Fig. 3A) despite decreased food intake (Supplementary Fig. 3B), whereas there were no significant differences in body composition, as assessed by nuclear magnetic resonance (NMR) (Supplementary Fig. 3C). Additionally, the expression of the thermogenic genes *Pgc-1α* (Fig. 4F) and *Prdm-16* (Fig. 4G) was lower in the brown adipose tissues of HFD-fed IL-9R KO mice upon long-term cold exposure, suggesting that reduced activation of thermogenic adipose tissues due to loss of IL-9R might be causative for the decreased energy expenditure of obese IL-9R KO mice.

### NLRP3 signaling mediates increased inflammation in HFD-fed IL-9R KO mice

**Fig. 5:**
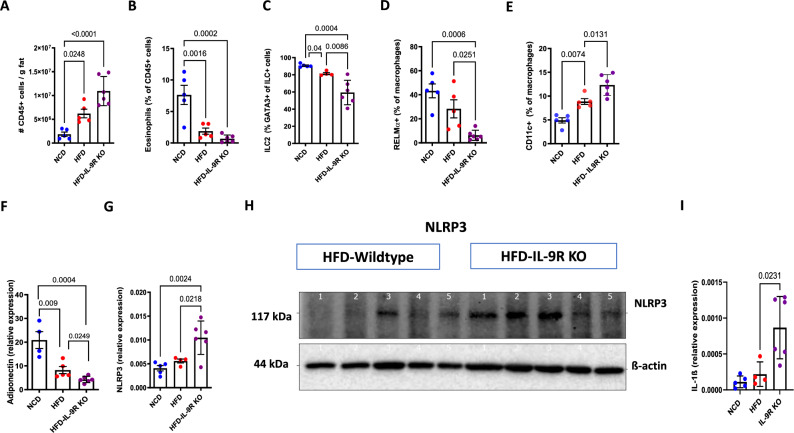
IL-9R deficiency leads to an increased inflammatory profile during diet-induced obesity. **A** Total immune cells per gram of fat; frequencies of **B** eosinophils, **C** ILC2s, **D** RELMα+ macrophages, **E** CD11c+ macrophages; gene expression of **F** adiponectin and **G** NLRP3; **H** protein expression of NLRP3; **I** gene expression of IL-1β in the adipose tissue of wild-type and IL-9R KO mice fed a HFD for 12 weeks. All experiments were repeated twice, and representative data are shown. One-way ANOVA was performed

**Fig. 6:**
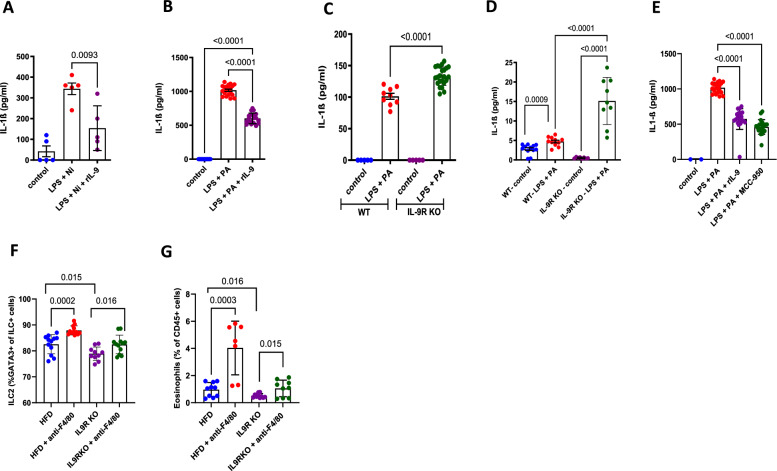
IL-9R deficiency leads to inflammasome-induced IL-1β release. Bone marrow-derived macrophages (BMDMs) were pretreated with recombinant IL-9 for 24 h and then treated with **A** LPS for 3.5 h and nigericin for 1 h; **B** LPS for 3 h and palmitic acid for 18 h, and the IL-1β levels in the supernatants were measured via ELISA. **C** The stromal vascular fraction and **D** adipose tissue macrophages were purified via FACS sorting from WT and IL-9R KO mice fed a HFD and treated with LPS for 3 h and palmitic acid for 18 h, and the IL-1β levels were measured via ELISA. **E** BMDMs were pretreated with rIL-9 or MCC-950 for 24 h and then with LPS for 3 h and palmitic acid for 18 h, and the IL-1β levels were measured via ELISA. WT and IL-9R KO mice were treated with anti-F4/80 or PBS twice per week from 9–12 weeks of HFD, and the frequencies of **F** ILC2s and **G** eosinophils were assessed. All experiments were repeated twice; representative data are shown except for **F** and **G**, where pooled data are shown. One-way ANOVA was performed

Despite decreased adipose tissue weight, total leukocyte cell numbers were increased in the adipose tissue of IL-9R KO mice (Fig. 5A). In addition to the increased number of immune cells, we observed decreased frequencies of cell types that are present in the adipose tissue of lean individuals and associated with improved insulin sensitivity, i.e., eosinophils (Fig. 5B), ILC2s (Fig. 5C), and RELMα+ macrophages (Fig. 5D), and increased frequencies of CD11c+ macrophages (Fig. 5E) in the adipose tissue of IL-9R KO mice fed a HFD. Representative figures are shown in Supplementary Fig. 4A‒C and Supplementary Fig. 5‒6. In line with this, the gene expression of the insulin-sensitizing adipokine *adiponectin* (Fig. 5F) was decreased in the visceral adipose tissue of the IL-9R KO mice fed a HFD. This effect was accompanied by increased gene and protein expression (Fig. 5G, H) of the NLRP3 and IL-1β gene expression (Fig. 5I), but not increased TNF and IL-6 expression (data not shown), indicating specific modulation of the inflammasome pathway by the IL-9/IL-9R signaling axis. To investigate the effect of IL-9 signaling on NLRP3, bone marrow-derived macrophages (BMDMs) from WT mice were treated with LPS and nigericin to induce NLRP3 signaling, and analyzed if pretreatment with rIL-9 decreased the release of IL-1β and IL-18, the effector cytokines of the NLRP3 pathway. We observed significantly decreased IL-1β (Fig. 6A) and slightly reduced IL-18 (*p* < 0.1; Supplementary Fig. 7A) secretion after treatment with rIL-9. Furthermore, rIL-9 treatment decreased both IL-1β and IL-18 levels in BMDM cultures stimulated with LPS and palmitic acid (PA) (Fig. 6B and Supplementary Fig. 7B). In addition, in the in vitro cultured adipose tissue stromal vascular fraction from control and IL-9R KO mice, treatment with LPS and PA led to increased release of IL-1β (Fig. 6C) and IL-18 (Supplementary Fig. 7C) in the IL-9R KO mice compared with the controls. Furthermore, adipose tissue macrophages were isolated from HFD-fed WT and IL-9R KO mice, and upon stimulation with LPS and PA, increased release of IL-1β was observed (Fig. 6D). We also confirmed that IL-1β release occurs via the NLRP3 pathway, as treatment with MCC-950, an inhibitor of the NLRP3 pathway, led to decreased IL-1β release, thereby phenocopying the effect of rIL-9 (Fig. 6E). Furthermore, in vivo macrophage depletion resulted in increased frequencies of ILC2s (Fig. 6F) and eosinophils (Fig. 6G) in the adipose tissue of HFD-fed IL-9R KO mice compared with PBS-treated controls, indicating that macrophages are the major target cell types of IL-9 during obesity.

### Abrogation of NLRP3 signaling rescues metabolic impairment in IL-9R KO mice

**Fig. 7:**
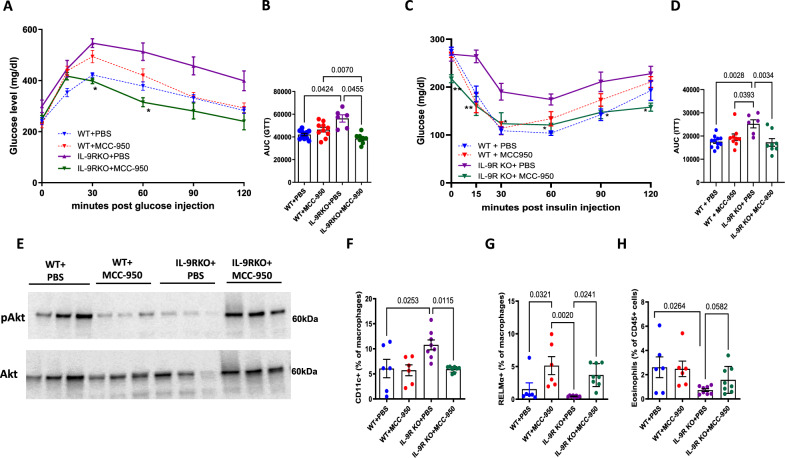
Inhibition of NLRP3 signaling improves metabolism and inflammation in IL-9R KO mice. **A** Glucose tolerance test (GTT); **B** AUC for GTT; **C** insulin tolerance test (ITT); **D** AUC for ITT; **E** expression of pAkt and total Akt 15 min after injection of insulin; frequencies of **F** CD11c+ macrophages, **G** RELM-α+ macrophages and **H** eosinophils in the adipose tissue of HFD-fed WT and IL-9R KO mice treated with PBS or MCC-950 thrice weekly from week 4 of HFD. All experiments were repeated twice; pooled data are shown for **A**–**D**, and representative data are shown. *p < 0.05, **p < 0.01 compared with the IL-9R KO + PBS group by two-way ANOVA. For **A** and **C**, two-way ANOVA was performed, and for the remaining, one-way ANOVA was performed

To explore the significance of our results in vivo, WT and IL-9R KO mice were fed a HFD and treated with MCC-950, a selective inhibitor of the NLRP3 inflammasome, beginning from week 4 of the HFD (50 mg/kg, 3x per week) until the end of the HFD at 12 weeks. Administration of MCC-950 resulted in a significant improvement in the GTT (Fig. 7A, B), ITT (Fig. 7C, D) and insulin-stimulated pAkt/Akt ratio (Fig. 7E) in HFD-fed IL-9R KO mice compared with their corresponding PBS treated HFD-fed IL-9R KO controls. Immunophenotyping of adipose tissue confirmed that MCC-950 treatment also resulted in decreased frequencies of classically activated CD11c+ macrophages (Fig. 7F) and increased frequencies of alternatively activated RELMα+ macrophages (Fig. 7G) and eosinophils (*p* = 0.058; Fig. 7H) in HFD-fed IL-9R KO mice compared with those in PBS-treated HFD-fed IL-9R KO controls. We also observed slightly increased visceral adipose tissue weight (Supplementary Fig. 8A) in the MCC-950-treated HFD-fed IL-9R KO mice compared with the PBS-treated HFD-fed IL-9R KO control mice, slightly reduced body weight (Supplementary Fig. 8B), no change in fasting glucose (Supplementary Fig. 8C) and decreased cholesterol (Supplementary Fig. 8D) levels. Taken together, the in vivo data showing improvements in HFD-fed IL-9R KO mice after MCC-950 treatment corroborate our in vitro findings that IL-9 signaling modulates NLRP3.

### IL-9 cooperates with IL-5 to inhibit NLRP3 signaling

As IL-9 has been previously shown to promote IL-5 [21] and IL-13 signaling [22], we next assessed whether other type 2 cytokines, such as IL-4, IL-13 and IL-5, are required for the observed effect of IL-9 on the inhibition of NLRP3 signaling. In agreement with these findings, we found that IL-9R KO mice presented decreased frequencies of IL-5 + IL-13 + CD4 + T cells (Supplementary Fig. 7D) and ILC2s (Supplementary Fig. 7E), indicating that IL-9 signaling is important for other type 2 cytokines. There were no differences in IL-4 + CD4 + T cells (Supplementary Fig. 7F) or ILC2s (Supplementary Fig. 7G). Treatment of WT SVF with rIL-9 increased IL-5 secretion (Supplementary Fig. 7H) but not IL-4 or IL-13 release (Supplementary Fig. 7I, J). However, in BMDMs, rIL-9 treatment did not increase the levels of any of these cytokines (data not shown), indicating that other cell types are the major sources of these cytokines, which is in agreement with our in vivo data. Next, using IL-4Rα/IL-5 KO mice, we assessed whether IL-4 and IL-5 signaling are necessary for the observed ability of rIL-9 to reduce IL-1β and IL-18 levels. First, in the SVF of IL-4Rα/IL-5 KO mice, treatment with rIL-9 did not reduce IL-1β levels (Supplementary Fig. 7K), as in WT mice. Furthermore, in BMDMs from IL-4Rα/IL-5 KO mice, rIL-9 treatment did not reduce IL-18 levels (Supplementary Fig. 7L) in IL-4R/IL-5 KO mice. However, the IL-4Rα/IL-5 KO mice generally presented lower IL-1β and IL-18 concentrations. Taken together, these findings suggest that IL-9 signaling functions in coordination with IL-5 signaling to reduce the levels of IL-1β and IL-18. Furthermore, IL-2Rγ was found to be important for IL-9 signaling, as observed in BMDMs and SVFs from Rag2IL-2Rγ KO mice. rIL-9 treatment did not reduce IL-1β (Supplementary Fig. 8E) or IL-18 (Supplementary Fig. 8F) levels in the BMDMs or the IL-1β (Supplementary Fig. 8G) levels in the SVFs of these mice.

### IL-9 acts via STAT5 to inhibit NLRP3 signaling

Since IL-9 signaling involves the activation of the STAT1, STAT3, and STAT5 proteins [37], we next determined the specific IL-9-induced STAT proteins involved in the regulation of the NLRP3 pathway. For this purpose, BMDMs from WT animals were cultured in the presence of LPS and PA, which increased IL-1β and IL-18 release, and as observed earlier, treatment with rIL-9 decreased IL-1β (Supplementary Fig. 9A). However, in the presence of the STAT5 inhibitor, rIL-9 was not able to decrease IL-1β (Supplementary Fig. 9A). Additionally, rIL-9 treatment decreased IL-18 (Supplementary Fig. 9B) but not in the presence of STAT1 and STAT5 inhibitors, demonstrating that the action of rIL-9 requires STAT5 and, to a lesser degree, STAT1. In addition, the expression of phospho-STAT5 (Supplementary Fig. 9C) and phospho-STAT1 (Supplementary Fig. 9D) was also increased after treatment with rIL-9. We also observed a decrease in the basal levels of phospho-STAT5 (Supplementary Fig. 9E) and phospho-STAT1 (Supplementary Fig. 9F) in the BMDMs of the IL-9R KO mice compared with those in the BMDMs of the WT controls. We detected decreased mRNA expression of NLRP3 after treatment with rIL-9. In response to treatment with the STAT5 inhibitor, this effect was abolished (Supplementary Fig. 9G), which is in agreement with the results of other studies, which revealed the transcriptional regulation of NLRP3 by STAT5 [38, 39].

### IL-9 is partially responsible for the effect of LsAg

**Fig. 8:**
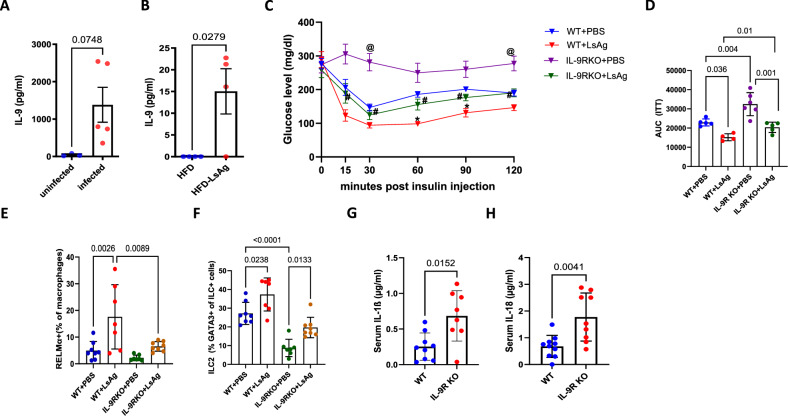
The metabolic improvements induced by helminth infection during diabetes are partially dependent on IL-9. **A** IL-9 levels in the pleura of naïve and *L. sigmodontis*-infected mice at 30 days post-infection (dpi); **B** plasma IL-9 levels of mice treated with PBS or filarial antigen (LsAg) for 14 days after 12 weeks of high-fat diet (HFD); **C** insulin tolerance test (ITT), **D** AUC for ITT; frequencies of **E** RELMα+ macrophages and **F** ILC2s in PBS- and LsAg-treated WT and IL-9R KO mice fed a HFD for 12 weeks. Serum **G** IL-1β and  **H** IL-18 levels in WT and IL-9R KO mice infected with *L. sigmodontis* at 35 dpi. All experiments were repeated twice, and representative data are shown. @*p* < 0.05 vs WT + PBS; #*p* < 0.05 vs IL-9R KO + PBS; *p < 0.05 vs WT + PBS. For **C** two-way ANOVA, **D**–**F** one-way ANOVA and for the rest, two-tailed unpaired *t* tests were performed

Considering the host protective role of IL-9 during helminth infections and the proven ability of *L. sigmodontis* infection and filarial antigen (LsAg) administration to improve glucose and insulin tolerance by inducing a regulatory, type 2 phenotype [33], we proceeded to identify whether the LsAg-mediated protection observed in diet-induced obesity, as observed in our earlier studies, is dependent on IL-9. We began by investigating IL-9 levels in the pleura of naïve and *L. sigmodontis*-infected mice at 30 days post-infection (dpi), a stage where fourth-stage larvae molt into adult worms. We detected significantly elevated levels of IL-9 in the pleura at 30 dpi in the *L. sigmodontis*-infected mice compared with the corresponding controls (Fig. 8A). Further treatment of HFD-fed mice with LsAg for 14 days also resulted in increased IL-9 levels in the plasma compared with PBS treated control mice (Fig. 8B). To determine whether the protective effect of LsAg is abolished in IL9R KO mice, we assessed glucose and insulin tolerance and adipose tissue inflammation in HFD-fed IL-9R KO mice treated with LsAg or PBS for 2 weeks daily. LsAg treatment improved insulin tolerance in both WT and IL-9R KO mice; however, the LsAg-treated WT mice had better insulin tolerance (Fig. 8C, D) than the LsAg-treated IL-9R KO mice did. In addition, the frequencies of RELMα+ macrophages (Fig. 8E) and ILC2s (Fig. 8F) were lower in the LsAg-treated IL-9R KO mice compared with the WT mice treated with LsAg. These findings indicate that IL-9 might support the effect of LsAg on glucose metabolism and adipose tissue inflammation. Notably, the serum IL-1β and IL-18 levels were increased at 35 dpi in the IL-9R KO mice, suggesting that the inflammasome was modulated during *L. sigmodontis* infection (Fig. 8G, H).

## Discussion

Despite the proven role of IL-9 in inflammation, resolution and tolerance during autoimmunity, allergy and parasitic infections, its role as a possible mediator of inflammation during metabolic diseases has been largely underexplored. Given the established role of adipose tissue inflammation in the pathogenesis of insulin resistance [40] and the emerging role of immune cell types in mediating metabolic homeostasis [6, 41], we investigated the role of the IL-9 signaling pathway in adipose tissue inflammation. Although there are two reports describing IL-9 levels in type 2 diabetes patients, these reports are inconclusive, with one showing decreased IL-9 levels [10] and another demonstrating increased IL-9 levels in diabetes patients [42]. Furthermore, these studies do not shed light on the possible downstream mechanistic pathways regulated by IL-9 signaling. In our human cohort, we found decreased IL-9 levels in nonfilaria-infected patients with diabetes compared with the corresponding controls without diabetes. We also found a negative correlation of IL-9 levels with body fat and CRP levels and a positive correlation with insulin levels and muscle mass. Treatment of human monocyte-derived macrophages with rIL-9 also decreased IL-1β levels. The findings in our animal model suggest that HFD-fed IL-9R KO mice have greater weight gain, impaired glucose and insulin tolerance, increased inflammation and decreased energy expenditure. During diet-induced obesity in mice, the frequencies of IL-9 + CD4 + T cells and ILC2s and IL-9R expression on macrophages are significantly decreased. Mechanistically, we further identified the inhibition of the NLRP3 inflammasome by the IL-9 signaling pathway in macrophages via in vitro and in vivo experiments. Moreover, in adipocytes, IL-9 signaling modulated adiponectin and TNF levels, suggesting the involvement of additional pathways. Furthermore, in this report, we show that helminth-mediated protection against obesity and insulin resistance is partially dependent on the IL-9 signaling pathway.

The administration of rIL-9 improved weight gain and insulin tolerance and increased eosinophil, RELMα+ macrophage and ILC2 frequencies in the adipose tissue of HFD-fed WT mice, which prompted us to work with IL-9R KO mice. Based on the reversal of changes observed in immune cell phenotypes, we hypothesized that adipose tissue inflammation could be the major mediator of obesity and the insulin resistance phenotype. In line with the results of rIL-9 administration, we found that HFD-fed IL-9R KO mice presented increased weight gain, impaired glucose and insulin tolerance, and decreased frequencies of eosinophils, RELMα+ macrophages and ILC2s in adipose tissue. We also found increased gene and protein expression of the NLRP3 inflammasome in the adipose tissue of HFD-fed IL-9R KO mice. Although NLRP3 is a well-known instigator of type 2 diabetes, the possibility that the IL-9 pathway could exert its protective effects by regulating the NLRP3 inflammasome has not been investigated thus far. To determine how IL-9 inhibits the NLRP3 inflammasome, we first conducted in vitro experiments in BMDMs. rIL-9 treatment decreased IL-1β and IL-18 secretion when BMDMs were stimulated via the inflammasome using a combination of LPS plus nigericin and LPS plus palmitic acid, the most abundant fatty acid in the body under obese and steady-state conditions. We were able to confirm these findings in the stromal vascular fraction as well as adipose tissue macrophage cultures of WT and IL-9R KO obese mice, wherein cultures from IL-9R KO mice showed increased IL-1β and IL-18 secretion. Treatment of BMDM cultures with an inhibitor of the NLRP3 inflammasome, MCC-950, mirrored the effects of rIL-9 in decreasing IL-1β, indicating that IL-1β secretion is indeed from the NLRP3 pathway. To confirm our findings in vivo, IL-9R KO mice fed a HFD were treated i.p. with MCC-950. The results of this experiment demonstrated that MCC-950 treatment improved glucose tolerance and insulin tolerance, improved the pAkt/Akt ratio, and decreased body weight and plasma cholesterol levels. Furthermore, MCC-950 treatment resulted in decreased frequencies of CD11c+ macrophages and increased frequencies of RELMα+ macrophages and eosinophils in the adipose tissue of HFD-fed IL-9R KO mice compared with the corresponding PBS-treated controls.

Taken together, our findings show that IL-9 signaling is protective against obesity and insulin resistance and that IL-9 mediates its effects on glucose homeostasis and inflammation by inhibiting the NLRP3 inflammasome. While demonstrating a novel mechanism by which IL-9 might mediate metabolic homeostasis and inflammation during insulin resistance and obesity, this study does not rule out further unidentified mechanistic roles for IL-9 signaling in mediating its effects. To date, there are no reports showing a possible interaction between the IL-9 pathway and the inflammasome in type 2 diabetes. However, in other settings, such as candidiasis, IL-9 has been shown to play a proinflammatory role by promoting NLRP3 inflammasome activity in the early stages of infection and, in the late stages, a tolerogenic role by promoting IL-1Rα production [43]. In agreement with other reports, IL-9 requires IL-2Rγ for its effects and seems to act in coordination with IL-5 to mediate its effect on IL-1β and IL-18 inhibition. In allergic airway inflammation, IL-9 has been shown to inhibit the expansion of alveolar macrophages [44].

IL-9 signal transduction is known to be mediated by the phosphorylation of the STAT1, STAT3 and STAT5 proteins, which form homo or heterodimers and translocate to the nucleus to regulate the expression of inflammatory pathways [45]. In BMDMs, we observed that rIL-9 was not able to inhibit IL-1β or IL-18 earlier in the presence of STAT5 inhibitors and, to a lesser degree, in the presence of a STAT1 inhibitor. In line with these results, the expression of phosphorylated STAT1 and STAT5 was decreased in the BMDMs of the IL-9R KO mice compared with those of the WT controls. Furthermore, treatment of WT BMDM cultures with LPS and palmitic acid decreased the expression of phosphorylated STAT1 and STAT5, and rIL-9 treatment increased the expression of phosphorylated STAT1 and STAT5. Taken together, these findings indicate that STAT1 and STAT5 are the major downstream components of the IL-9 pathway in orchestrating its effects on the NLRP3 inflammasome. In line with our results, an earlier report showed that the treatment of human macrophages with rIL-9 increased the activation of STAT5 and STAT1 [46]. We also observed decreased mRNA expression of NLRP3 in response to rIL-9 treatment, which was found to be STAT5 dependent. Further experiments, such as chip sequencing, to determine whether STATs bind to the NLRP3 promoter to inhibit its expression could provide conclusive answers to how IL-9 inhibits NLRP3 signaling. Interestingly, an earlier report showed that STAT5 controls the expression of NLRP3 [39], and in a recent report, STAT5 was shown to inhibit NLRP3-induced pyroptosis [38].

While visceral adipose tissue is a hub of low-grade inflammation during obesity and diabetes development, brown adipose tissue is the energy dissipating organ that decreases with weight gain and aging. Thus, we investigated energy expenditure defects in IL-9R KO mice upon exposure to cold. Compared with WT control conditions, acute and long-term cold exposure led to decreased O_2_ consumption in HFD-fed IL-9R KO mice, indicating decreased energy expenditure. Throughout cold exposure, IL-9R KO mice tended to remain obese despite decreased food intake. Body composition measurements, including fat-free mass and muscle mass, were not altered in the IL-9R KO mice compared with the corresponding controls. We did not assess whether MCC-950 treatment could rescue defects in energy expenditure in IL-9R KO mice in the present study. However, this possibility cannot be ruled out, as NLRP3 has already been shown to be involved in postburn-induced lipolysis [47]. On the other hand, it is possible that IL-9 affects energy expenditure via other unknown mechanisms.

Finally, we investigated the role of IL-9 in helminth-mediated protection during type 2 diabetes. Previous studies from our group have shown that the administration of LsAg to HFD-fed mice improved glucose tolerance, increased the frequencies of RELMα+ macrophages, eosinophils, and ILC2s and decreased the number of classical CD11c+ macrophages in adipose tissue [33]. We also tested whether the effects of LsAg are mediated by IL-9. Upon the administration of LsAg to WT and IL-9R KO mice, we observed that IL-9R KO mice presented impaired insulin tolerance compared with WT-HFD-fed mice treated with LsAg. Similarly, there was a less pronounced shift in RELMα+ macrophages and ILC2s on LsAg treatment in the IL-9R KO mice than in the LsAg-administered WT controls. Furthermore, in animals infected with *L. sigmodontis*, the plasma levels of IL-1β and IL-18 were increased in the IL-9R KO mice, indicating increased NLRP3 levels during IL-9R deficiency. In summary, our results  signify a role for IL-9 in mediating the protective effects of LsAg on diabetes.

In summary, our data reveal a novel role for IL-9 signaling in the regulation of the NLRP3 inflammasome and thereby protection against impaired metabolic changes and inflammation. In addition to these changes, IL-9R deficiency also led to decreased energy expenditure during acute and long-term cold exposure, which may or may not be dependent on the increased inflammation induced by IL-9 deficiency. In addition, we also demonstrate a role for IL-9 in the protective effects of helminth infection during obesity and insulin resistance.

## Methods

### Human subjects

Serum samples were obtained from 159 participants, 83 of whom were infected with the filarial nematodes *Onchocerca volvulus (48.2%*) and/or *Mansonella perstans (54.2%*) and/or *Loa loa (47%*) from the littoral regions of Cameroon. The samples were obtained from a larger study designed to assess the associations of *O. volvulus*, *M. perstans* and *L. loa* with diabetes incidence in Cameroon. The detailed methodology of the study has been published elsewhere [48]. This study was designed as a partially controlled, open-label pilot trial to investigate the impact of filarial infections on metabolic and immunological profiles. The samples were from several scouted sites in rural areas around the littoral region of Cameroon, and the study was not a randomized controlled trial. On the basis of the HbA1c levels, 85 participants were classified as nondiabetic, and 74 were classified as diabetic. Among the nondiabetic subjects, 47 had a filarial infection, and 38 were endemic normal individuals. In the diabetic group, 36 patients were infected, and 38 were not infected. A clinical assessment of all the subjects was performed upon enrollment, and parameters, including sex, age, and BMI, were collected via standard procedures. HbA1c, fasting blood glucose, cholesterol, triglyceride, low-density lipoprotein (LDL) and high-density lipoprotein (HDL) levels were assessed via a HumaStar200 autoanalyzer (Human Biochemica und Diagnostica GmbH, Wiesbaden, Germany), and current medication use was recorded. The levels of IL-9 in the sera of patients were measured via Luminex ProcartaPlex assays (Thermo Fisher Scientific, Waltham, Massachusetts, USA) according to the manufacturer’s instructions in a blinded fashion. For further analysis, type 2 diabetes was diagnosed using an HbA1c value of ≥48 mmol/mol as per the WHO and ADA guidelines, whereas participants with an HbA1c value < 48 mmol/mol were considered nondiabetic controls. Ethical approval for the human study was obtained from the Ethics Committees at University Hospital, Bonn, Germany, and the Cameroon National Ethics Committee, and all participants provided written, informed consent.

### Animals and animal care

The experiments were performed using male wild-type (WT) and IL-9R KO mice on a C57BL/6 J background. WT C57BL/6 J mice were purchased from Charles River Labs. IL-9R KO mice were originally generated by Prof. Dr. Jean Christoph Renauld (Université Catholique de Louvain–Belgium) and were obtained from Prof. Minka Breloer, Bernhard-Nocht Institute for Tropical Medicine, Hamburg, Germany. IL-4R-IL-5KO BALB/c mice were originally obtained from Prof. Dr. Klaus Matthaei (Matthaei, Stem Cell & Gene Targeting Laboratory, ANU College of Medicine, Biology and Environment, Canberra, Australia). Rag2IL-2Rγ KO C57BL/6 J mice were bred in house. The mice were bred and housed at the animal facilities of the Institute for Medical Microbiology, Immunology and Parasitology (IMMIP), University Hospital Bonn, Germany. The mice were maintained in individually ventilated cages with a 12-h day/night cycle and food and water ad libitum. Starting at 6–8 weeks of age, subsets of mice were fed a high-fat diet (HFD), which provides 60% of calories from fat for 10–12 weeks (Research Diets, Inc., New Brunswick (NJ), USA).

### Glucose tolerance test (GTT) and insulin tolerance test (ITT)

After 6 h of fasting, the mice were injected intraperitoneally (i.p.) with 1 g of glucose solution per kilogram of body weight. Blood glucose levels were measured from tail vein blood via a blood glucose meter (Accu-Check Advantage; Roche Diagnostics GmbH, Mannheim, Germany) immediately before and 15, 30, 60, 90, and 120 min after glucose injection. The area under the curve (AUC) was obtained by calculating the area between the x-axis and a given curve. For the insulin tolerance test, after 4 h of fasting, insulin (1 U/kg body weight) was administered intraperitoneally, and blood glucose levels were measured immediately before and 15, 30, 60, 90, and 120 min after insulin injection.

### Indirect calorimetry and body composition

For metabolic characterization of WT and IL-9R KO mice, oxygen consumption, carbon dioxide production and motility were measured via the Phenomaster system (TSE Systems) with a light and dark cycle of 12 h each. The animals were singly caged with access to food and water ad libitum unless otherwise noted. Upon introduction into the system, the animals were allowed to acclimatize for 48 h at 23 °C, of which the final 24 h were taken as the baseline measurement. Next, acute cold exposure was performed for 2 h at 4 °C during the light cycle without access to food and water, after which the animals were allowed to regenerate for 48 h at 23 °C. Long-term cold exposure was subsequently initiated for 3 days at 16 °C, followed by 7 days at  4°C. For each cycle, each animal was measured for 2 min. The position of the animals within the Phenomaster system was randomized. The animals and food were weighed once per day around noon. Body composition was analyzed at the end (day 10) of long-term cold exposure via a Bruker Minispec LF50H.

### In vivo inhibitor treatment

To block the NLRP3 inflammasome, 50 mg/kg MCC950 inhibitor (MedChem Express, Sollentuna, Sweden) was dissolved in sterile PBS and administered i.p. to HFD-fed WT and IL-9R KO mice thrice weekly beginning at week 5 of HFD. The control mice received PBS injections. For rIL-9 treatment, PBS or 2 µg/kg rIL-9 thrice weekly was administered i.p. to HFD- or NCD-fed mice.

### LsAg preparation and administration

Crude *Litomosoides sigmodontis* adult worm extract (LsAg) was prepared as described previously [49]. Briefly, *L. sigmodontis* adult worms were harvested from the thoracic cavity of infected gerbils (*Meriones unguiculatus*) and mechanically homogenized on ice in endotoxin-free PBS (PAA, Pasching, Austria) via a sterile glass potter. Following centrifugation at 3200 × *g*, the supernatant was collected, and the protein concentration was measured via the Bradford assay (Cytoskeleton, Denver, USA). Aliquots of LsAg were stored for later use at −80°C. Daily i.p. injections of 2 µg of LsAg per mouse for 2 weeks were given to male C57BL/6 J diet-induced obesity (DIO) mice during weeks 8–10 or 12–14 of HFD. The controls received equal amounts of sterile PBS. After the final LsAg injection, all groups of mice were subjected to glucose and insulin tolerance tests, and immunological studies were performed 1 week later.

### Isolation of the stromal vascular fraction from visceral adipose tissue and flow cytometry

Visceral fat pads were diced and digested with 0.25 mg/ml Liberase TL (Roche, England) and 1 mg/ml DNase I (Sigma, St. Louis, USA) at 37 °C for 25 min. The digested tissue was washed, ground and filtered through 100 µm and 40 µm filters. The isolated cells were subjected to lysis of red blood cells with ammonium-chloride-potassium (ACK) lysis buffer. Single-cell suspensions were stained with fluorochrome-conjugated antibodies against the following surface antigens: CD45-APCFire, CD11b-PEcy7, CD11c-BV605, SiglecF-PE, F4/80-PerCp-Cy5.5, CD90.2-BV605, CD4-AF700, CD4-BV785, IL-33R-PEcy7, IL-9R-FITC, Lineage Cocktail-Pacific blue, NK1.1-AF647, CD3-AF700, CD19-BV421, Ly6G-FITC, and Ly6G-BV421 (BioLegend, California, USA; BD Biosciences, New Jersey, USA; Thermo Fisher Scientific, Massachusetts, USA). For examination of transcription factors, cells were subsequently treated with a FoxP3 fixation/permeabilization kit (eBioscience, California, USA) in accordance with the manufacturer’s instructions and stained for 30 min on ice with GATA3-AF647 and RELMα-APC antibodies. For intracellular cytokine staining, the cells were stimulated for 3 h with phorbol 12,13-dibutyrate (0.5 mg/ml) (PdBU; Biomol, Hamburg, Germany) and ionomycin (0.5 mg/ml) (Sigma, St. Louis, USA) in the presence of brefeldin A (1 mg/ml) (GolgiPlug, BD Biosciences, New Jersey, USA). Stimulated cells were stained for surface markers, fixed and permeabilized with a FoxP3 fixation/permeabilization kit (eBioscience, California, USA) in accordance with the manufacturer’s instructions, and stained with antibodies. IL-9-PE, IL-9-AF647, IL-5-PE, IL-4-Percp.cy5.5, and IL-13-AF488 (BioLegend, California, USA; BD Biosciences, New Jersey, USA; Thermo Fisher Scientific, Massachusetts, USA) were the antibodies used for intracellular cytokine staining. Phospho-STAT1 and phospho-STAT5 (BioLegend, California, USA) staining was performed via methanol permeabilization.

### Fluorescence-activated cell sorting and in vitro culture of adipose tissue macrophages

Macrophages were sorted by flow cytometry from adipose tissue stromal vascular fraction isolated from HFD-fed wild-type or IL-9R KO mice on the basis of positive selection for F4/80 and CD11b surface markers at the flow cytometry core facility of University Hospital, Bonn. Sort-purified macrophages were cultured as indicated.

### Western blotting

Visceral adipose tissue from WT and IL-9R KO mice fed a NCD or HFD for 12 weeks was homogenized and lysed in RIPA buffer (Thermo Fisher Scientific, Waltham, USA) for protein extraction. Protein separation was performed via SDS‒PAGE, and the proteins were transferred onto a nitrocellulose membrane. The membrane was incubated with primary antibodies against NLRP3, pAkt (ser-473), total Akt or β-actin (Cell Signaling Technology, Danvers, USA) overnight. The membrane was subsequently incubated with an HRP-conjugated secondary antibody (rabbit or mouse) (Cell Signaling Technology, Danvers, USA) for 1 h. PierceTM ECL Western Blotting Substrate (Thermo Fisher Scientific, Waltham, USA), the VersaDoc 5000 imaging system (Bio-Rad; Hercules, CA, USA), and the ImageJ program were used for detection, visualization and quantification, respectively.

### Real-time PCR

Tissues from WT and IL-9R KO mice fed a NCD or HFD were stored in RNAlater and subsequently stored at −80 °C (Thermo Fisher Scientific, Waltham, USA). Total RNA was extracted from the tissues via an RNeasy Plus kit (Qiagen, Hilden, Germany). cDNA conversion was performed via the Revert Aid First Strand cDNA Synthesis Kit (Thermo Fischer Scientific, Waltham, USA) according to the manufacturer’s instructions. The cDNA served as a template for amplification of the genes of interest. For analysis of genes expressed in adipose tissue, TaqMan probes for *adiponectin, nlrp3, pgc-1α, and prdm-16* (IDT technologies, Leuven, Belgium) were used, and target gene expression was calculated via the comparative method for relative quantification upon normalization to ß-actin gene expression.

### BMDM culture

For the culture of bone marrow-derived macrophages (BMDMs), the mice were euthanized, the hind legs were collected, the bone marrow was harvested and filtered, and red blood cell lysis was performed. Cell counts were determined with a CASY-cell counter. A total of 1 × 10^6^ cells/ml were seeded in advanced RPMI medium (GIBCO, Thermo Fisher Scientific Inc., Germany) supplemented with 10% fetal bovine serum (FBS) (GIBCO, Thermo Fisher Scientific Inc., Germany), 1% penicillin/streptomycin (GIBCO, Thermo Fisher Scientific Inc., Germany), 0.1% gentamycin (GIBCO, Thermo Fisher Scientific Inc., Germany), 2.5% HEPES (GIBCO, Thermo Fisher Scientific Inc., Germany), and 1% GlutaMAX (GIBCO, Thermo Fisher Scientific Inc., Germany) supplemented with 1 µg/ml M-CSF (Peprotech Inc., Germany). Half of the medium was exchanged on day 4. On day 8, the cells were harvested, and the macrophage purity was checked via flow cytometry. The purity of the macrophages was analyzed via anti-F4/80 and anti-CD11b antibodies, and flow cytometry was performed via CytoFLEX (Beckman Coulter Life Sciences, USA). The data were analyzed via FlowJo 10.4.2 software. The purity was always above 90%. 200 ng/ml LPS (Sigma, St. Louis, USA), 1 mM palmitic acid (Cayman Chemicals, Ann Arbor, USA), 10 µM nigericin (Selleck, Cologne, Germany), 10 µM fludarabine (STAT1 inhibitor) (Selleck, Cologne, Germany), 10 µM CAS 285986-31-4 (STAT5 inhibitor) (Calbiochem, Darmstadt, Germany), and 500 pg/ml recombinant mouse IL-9 (BioLegend, California, USA).

### White adipocyte isolation and differentiation

Primary white adipocytes were isolated from 8–12-week-old C57BL/6 or IL-9R KO mice and used to perform the experiments. Dissected visceral fat pads from 2–3 different mice were digested in DMEM (Invitrogen by Thermo Fisher Scientific, Waltham, USA) containing 0.5% BSA and collagenase type II at 37 °C and then centrifuged at 250 × *g* for 10 min. The resulting pellet was resuspended and filtered through 100 µm nylon mesh. The filtered solution was seeded in 12-well plates at a density of 80,000 cells per well in DMEM supplemented with 10% FBS and 1% penicillin–streptomycin (WA growth medium) and kept at 37 °C under 5% CO_2_. Then, 48 h after seeding, the cells were washed with PBS and maintained with WA growth medium at 37 °C and 5% CO_2_. The medium was changed every other day until the cells reached confluency. Once confluent, preadipocytes were induced (day 0 to day 4) by changing the medium to WA induction medium (DMEM containing 5% FBS, 1% penicillin–streptomycin, 1 nM T3, 0.172 µM insulin, 50 mg ml^−1^ L-ascorbate, 1 mM d-biotin, 17 mM pantothenate, 1 µM rosiglitazone, 1 µM dexamethasone and 0.5 mM 3-isobutyl-1-methylxantine (Sigma, St. Louis, USA)). From day 4 until day 8, the cells were maintained in WA maintenance medium (DMEM containing 5% FBS, 1% penicillin–streptomycin, 1 nM T3, 0.172 µM insulin, 50 mg ml^−1^ L-ascorbate, 1 mM d-biotin, and 17 mM pantothenate), which was replenished every other day. The cells were treated on day 8 with LPS (200 ng/ml), palmitic acid (500 µM) or rIL-9 (5 ng/ml).

### Human macrophage culture

Buffy coats were obtained from the blood bank of the University Hospital Bonn. Information regarding the sex and age of the donors was not supplied by the blood bank. The blood from the buffy coats was diluted 1:2 in PBS. PBMCs were isolated with a Pancoll gradient (Pan-Biotech, Aidenbach, Germany) and centrifuged at 2000 × *g* without breaks for 20 min. Next, peripheral blood mononuclear cells (PBMCs) were counted in the same manner as BMDMs and plated at a density of 2 × 10^6^ cells/ml in the appropriate Petri dish for each experiment in 20 ml of RPMI medium with 10% FBS (good forte, Pan-Biotech Aidenbach, Germany) supplemented with 2 mM L-glutamine, 100 U/ml penicillin, and 100 mg/ml streptomycin (full IMDM medium). After settling for 24 h, nonadherent cells were washed away with PBS, and the medium was replaced with full RPMI medium supplemented with 10% FCS and 50 ng/ml M-CSF (Miltenyi, Bergisch Gladbach, Germany) for 6 days of differentiation, resulting in >95% macrophage purity. On day 3, the medium was replaced with fresh medium supplemented with M-CSF. On day 6, the cells (near 100% macrophage purity) were detached with 1 mM EDTA and incubated for 30 min at 4 °C, followed by mild scraping. The cells were left untreated or stimulated in fresh medium without M-CSF. Recombinant human IL-9 (0.05 ng/ml-50 ng/ml, PeproTech, Thermo Fisher Waltham, USA) was used for the treatments.

### Macrophage depletion

F4/80^hi^ macrophages were depleted via the use of a monoclonal anti-F4/80 antibody (*InVivo*MAb anti-mouse F4/80, Bioxcell, West Lebanon, USA) during 9–12 weeks of high-fat feeding. The mice were intraperitoneally (i.p.) injected twice per week with 100 μg of the antibody or phosphate-buffered saline. The macrophage depletion efficiency was greater than 80%. For the macrophage depletion experiments, eosinophils were gated as CD45+SiglecF+ cells.

### ELISA

IL-1β, IL-18, TNF, IL-4, IL-5, IL-13 (Invitrogen by Thermo Fisher Scientific, Waltham, USA), and adiponectin (R&D Systems, Inc., Minneapolis, USA) levels were measured in the plasma and supernatants via ELISA according to the manufacturers’ instructions.

### Adipose tissue histology staining

Epididymal adipose tissue (EAT) was fixed in 4% paraformaldehyde (Otto Fischer GmbH, Germany) for at least 24 h. After fixation, the tissue was dehydrated with ethanol and then embedded in paraffin for sectioning. Hematoxylin‒eosin (HE) staining was performed following standard procedures.

### Statistics

The data were tested for statistical significance via GraphPad Prism software (version 9.5; GraphPad Software, San Diego, Calif., USA). Data that were normally distributed were tested for statistical significance via the unpaired *t* test for comparisons of two groups or the ANOVA test followed by the Newman‒Keuls multiple-comparison test for comparisons of more than two groups. Two-way ANOVA was used to evaluate the results of the GTT and ITT experiments. Values of *p* < 0.05 were considered statistically significant.

### Ethics statement

The animal housing conditions and procedures used in this work were performed according to the European Union animal welfare guidelines. All protocols were approved by the Landesamt für Natur, Umwelt und Verbraucherschutz, Cologne, Germany (84-02.04.2016. A331, 81-02.04.2020. A103 and 81-02.04.2022.A404).

## Supplementary information

Supplementary figures and tables

Supplementary figures and tables legends and caption
