# Supplementary material for: The role of the IL-9‒NLRP3 axis in insulin resistance and adipose tissue inflammation during diet-induced obesity
Source: Cell Mol Immunol. 2025 Sep 18;22(11):1478–90. doi: 10.1038/s41423-025-01340-4 (PMC12575696; doi:10.1038/s41423-025-01340-4)
Supplement: Supplementary file 1 — Supplementary figures and tables [file 41423_2025_1340_MOESM1_ESM.pdf]

Suppl. Fig.1

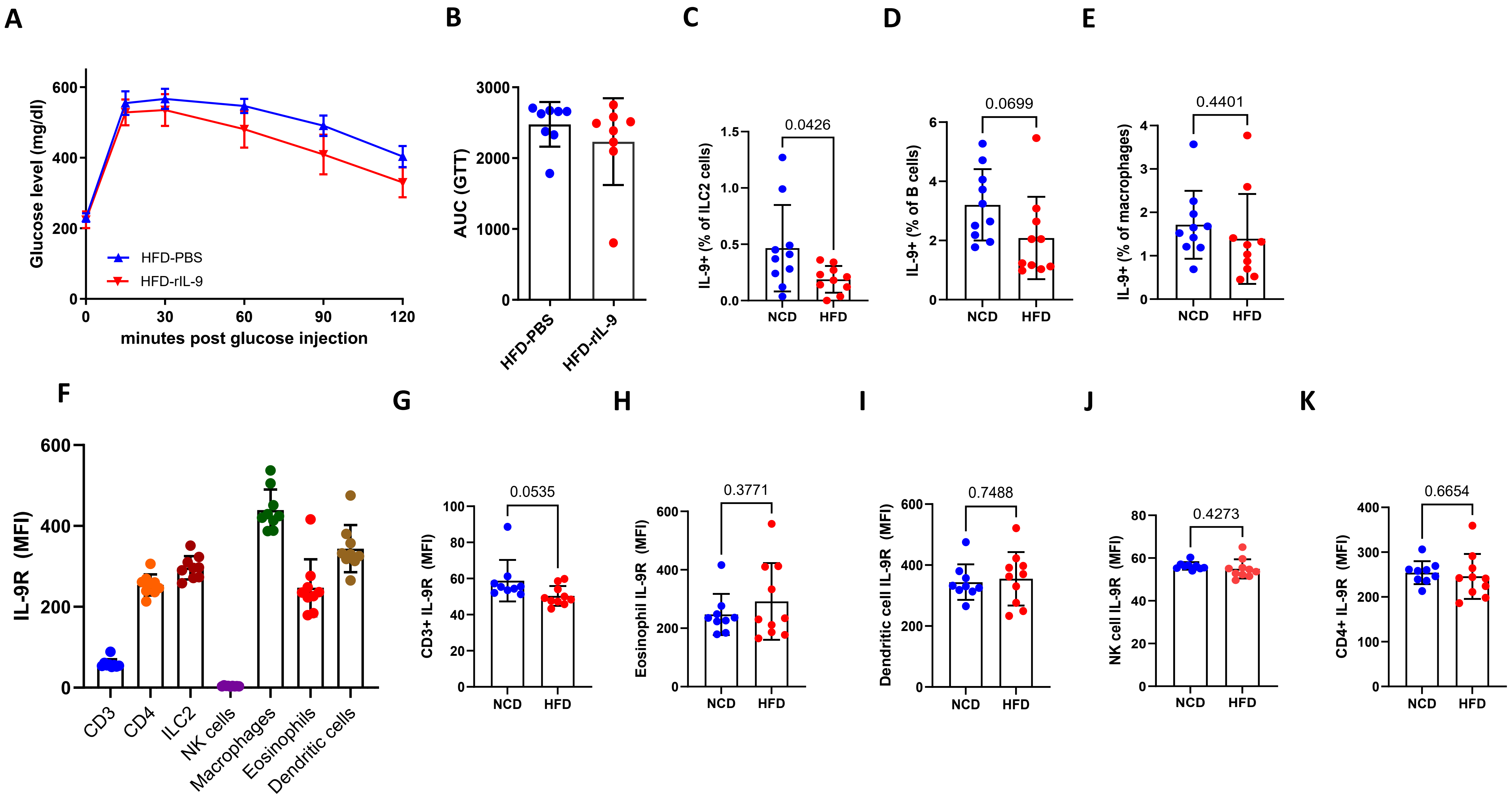

A
B
C
D
E
Glucose level (mg/dl)
minutes post glucose injection
HFD-PBS
HFD-rIL-9
AUC (GTT)
HFD-PBS
HFD-rIL-9
IL-9+ (% of ILC2 cells)
0.0426
IL-9+ (% of B cells)
0.0699
IL-9+ (% of macrophages)
0.4401
NCD
HFD
F
G
H
I
J
K
IL-9R (MFI)
CD3
CD4
ILC2
NK cells
Macrophages
Eosinophils
Dendritic cells
CD3+ IL-9R (MFI)
0.0535
Eosinophil IL-9R (MFI)
0.3771
Dendritic cell IL-9R (MFI)
0.7488
NK cell IL-9R (MFI)
0.4273
CD4+ IL-9R (MFI)
0.6654

Suppl. Fig.2

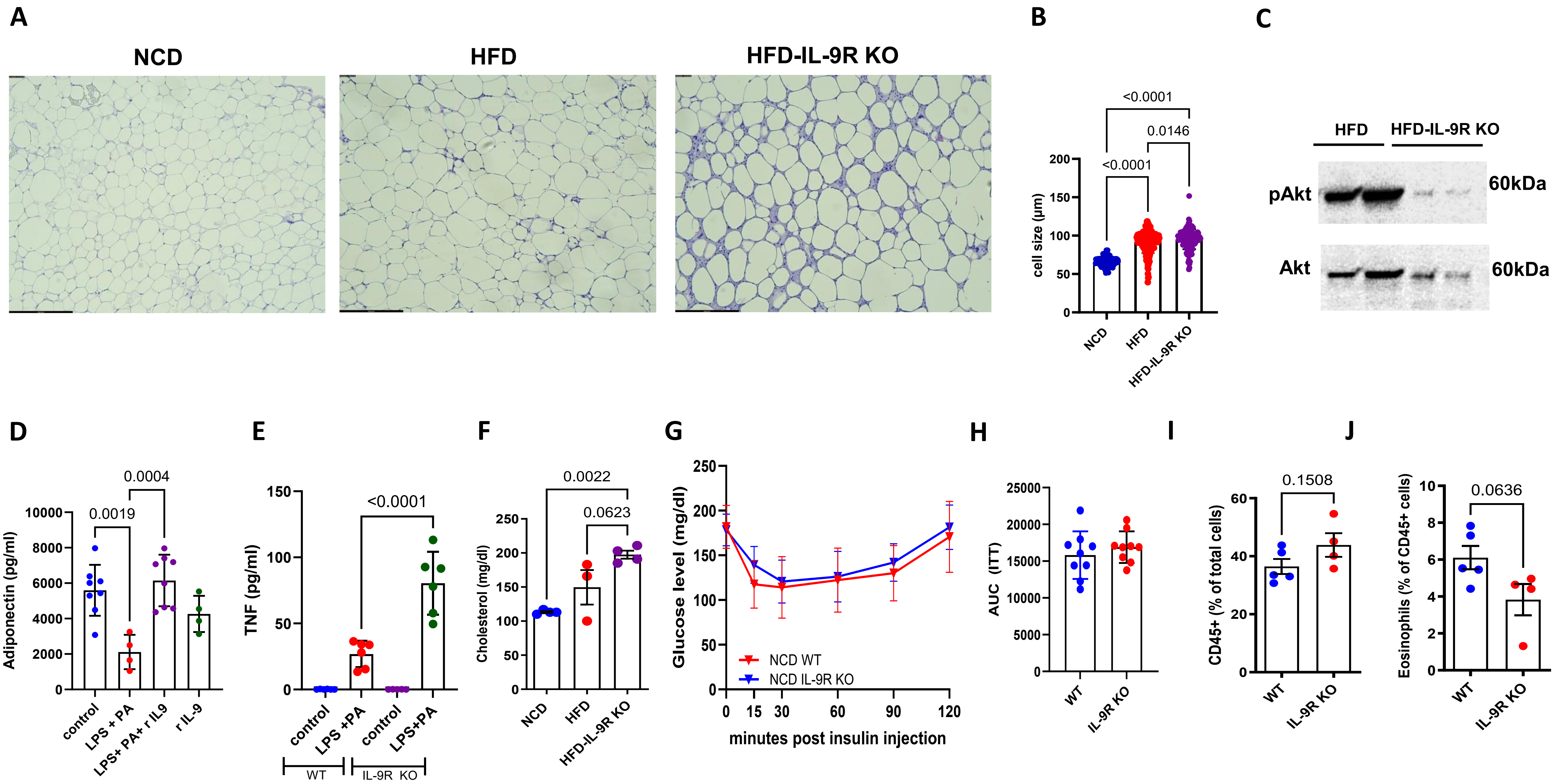
A
NCD
HFD
HFD-IL-9R KO
B
cell size (µm)
<0.0001
0.0146
<0.0001
NCD
HFD
HFD-IL-9R KO
C
HFD
HFD-IL-9R KO
pAkt
Akt
60kDa
60kDa
D
Adiponectin (pg/ml)
0.0019
0.0004
control
LPS + PA
LPS+ PA+ r IL9
r IL-9
E
TNF (pg/ml)
<0.0001
control
LPS +PA
control
LPS+PA
WT
IL-9R KO
F
Cholesterol (mg/dl)
0.0022
0.0623
NCD
HFD
HFD-IL-9R KO
G
Glucose level (mg/dl)
NCD WT
NCD IL-9R KO
minutes post insulin injection
H
AUC (ITT)
WT
IL-9R KO
I
CD45+ (% of total cells)
0.1508
WT
IL-9R KO
J
Eosinophils (% of CD45+ cells)
0.0636
WT
IL-9R KO

Suppl. Fig.3

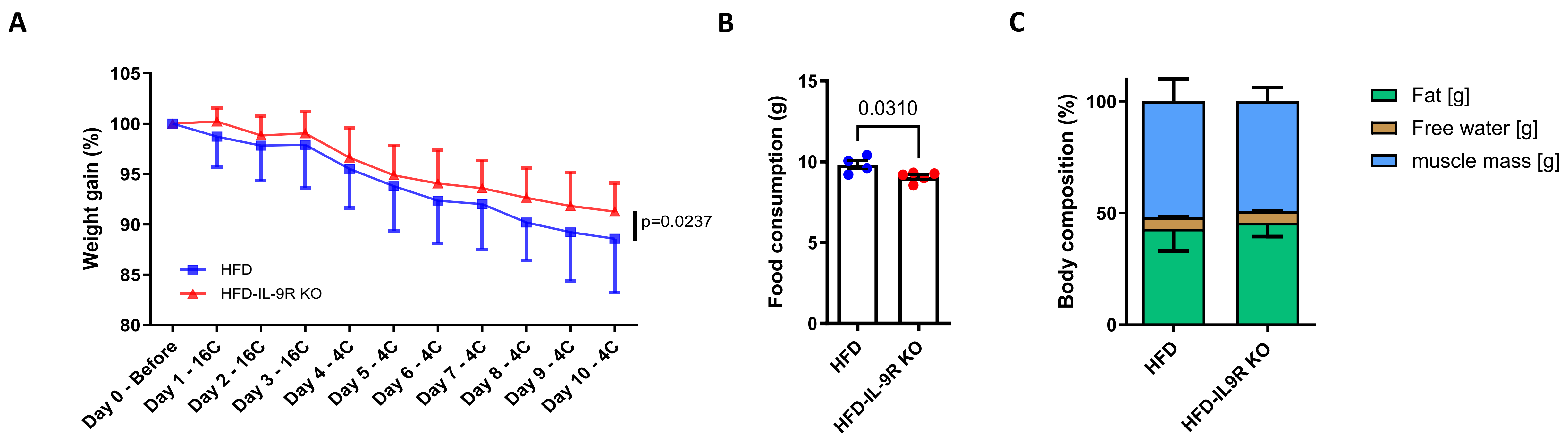
A
Weight gain (%)
105
100
95
90
85
80
HFD
HFD-IL-9R KO
p=0.0237
Day 0 - Before
Day 1 - 16C
Day 2 - 16C
Day 3 - 16C
Day 4 - 4C
Day 5 - 4C
Day 6 - 4C
Day 7 - 4C
Day 8 - 4C
Day 9 - 4C
Day 10 - 4C
B
Food consumption (g)
15
10
5
0
0.0310
HFD
HFD-IL-9R KO
C
Body composition (%)
100
50
0
Fat [g]
Free water [g]
muscle mass [g]
HFD
HFD-IL9R KO

Suppl.Fig.4

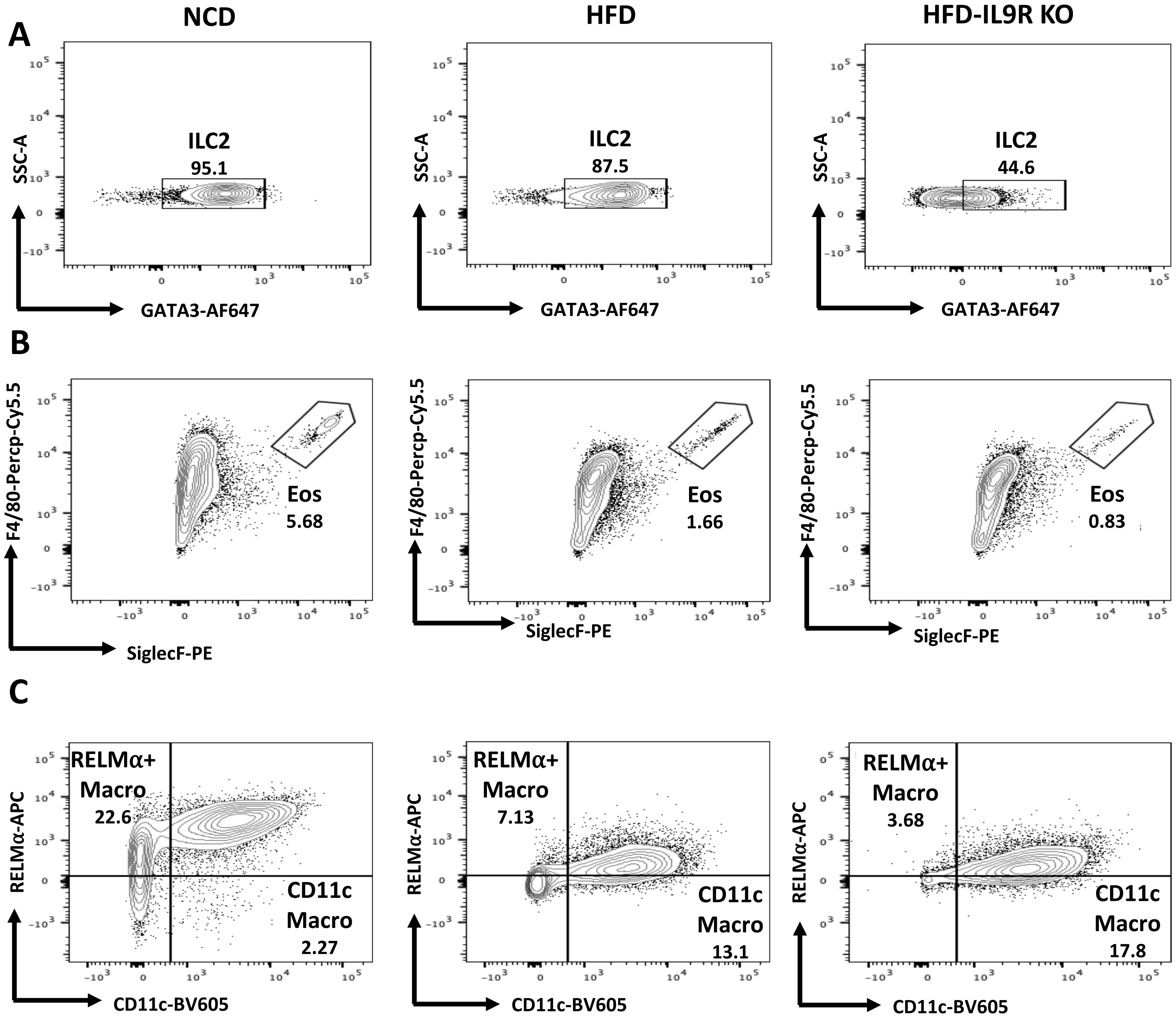
A
NCD
HFD
HFD-IL9R KO
ILC2
95.1
87.5
44.6
SSC-A
GATA3-AF647
B
F4/80-Percp-Cy5.5
SiglecF-PE
Eos
5.68
1.66
0.83
C
RELMα+
Macro
22.6
7.13
3.68
CD11c
Macro
2.27
13.1
17.8
RELMα-APC
CD11c-BV605

Suppl. Fig.5

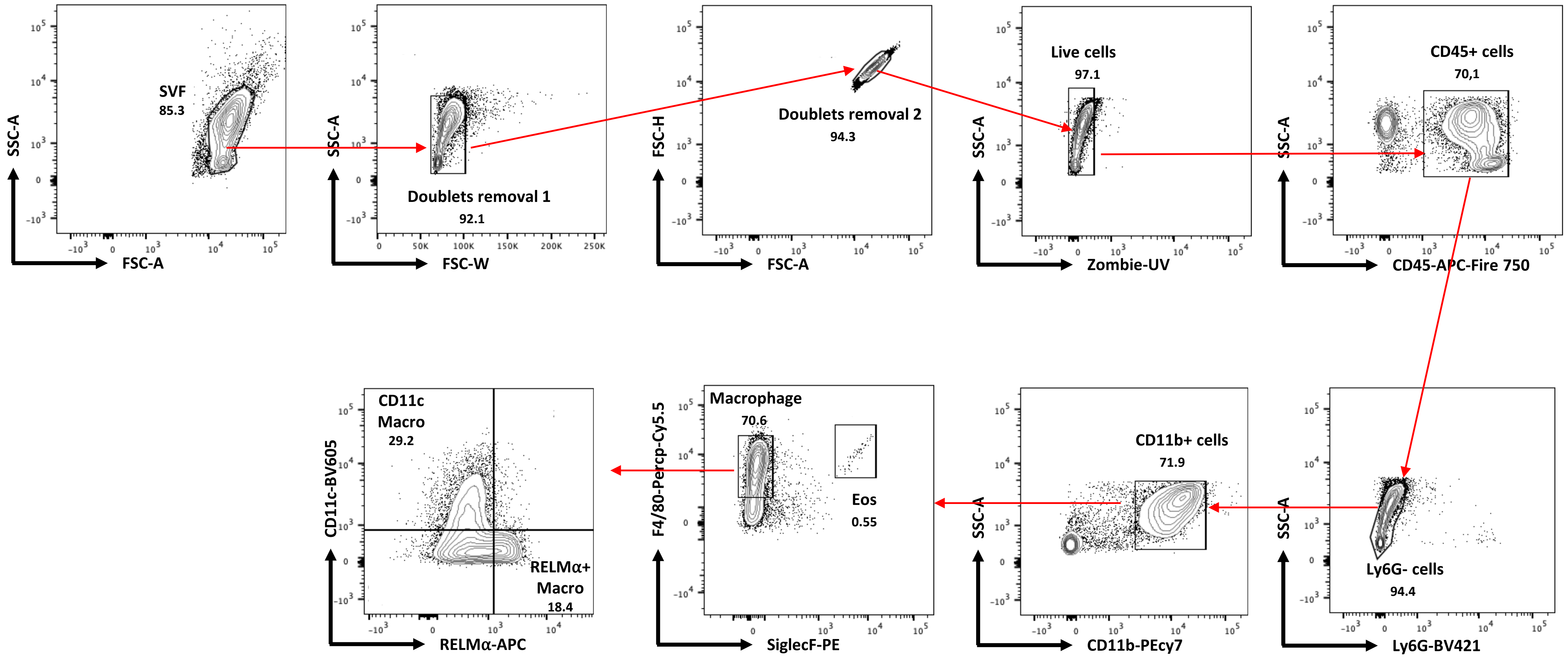

SVF
85.3
SSC-A
FSC-A
Doublets removal 1
92.1
FSC-W
Doublets removal 2
94.3
FSC-H
FSC-A
Live cells
97.1
SSC-A
Zombie-UV
CD45+ cells
70,1
SSC-A
CD45-APC-Fire 750
Ly6G- cells
94.4
SSC-A
Ly6G-BV421
CD11b+ cells
71.9
SSC-A
CD11b-PEcy7
Macrophage
70.6
Eos
0.55
F4/80-Percp-Cy5.5
SiglecF-PE
CD11c
Macro
29.2
RELMα+
Macro
18.4
CD11c-BV605
RELMα-APC

Suppl. Fig.6

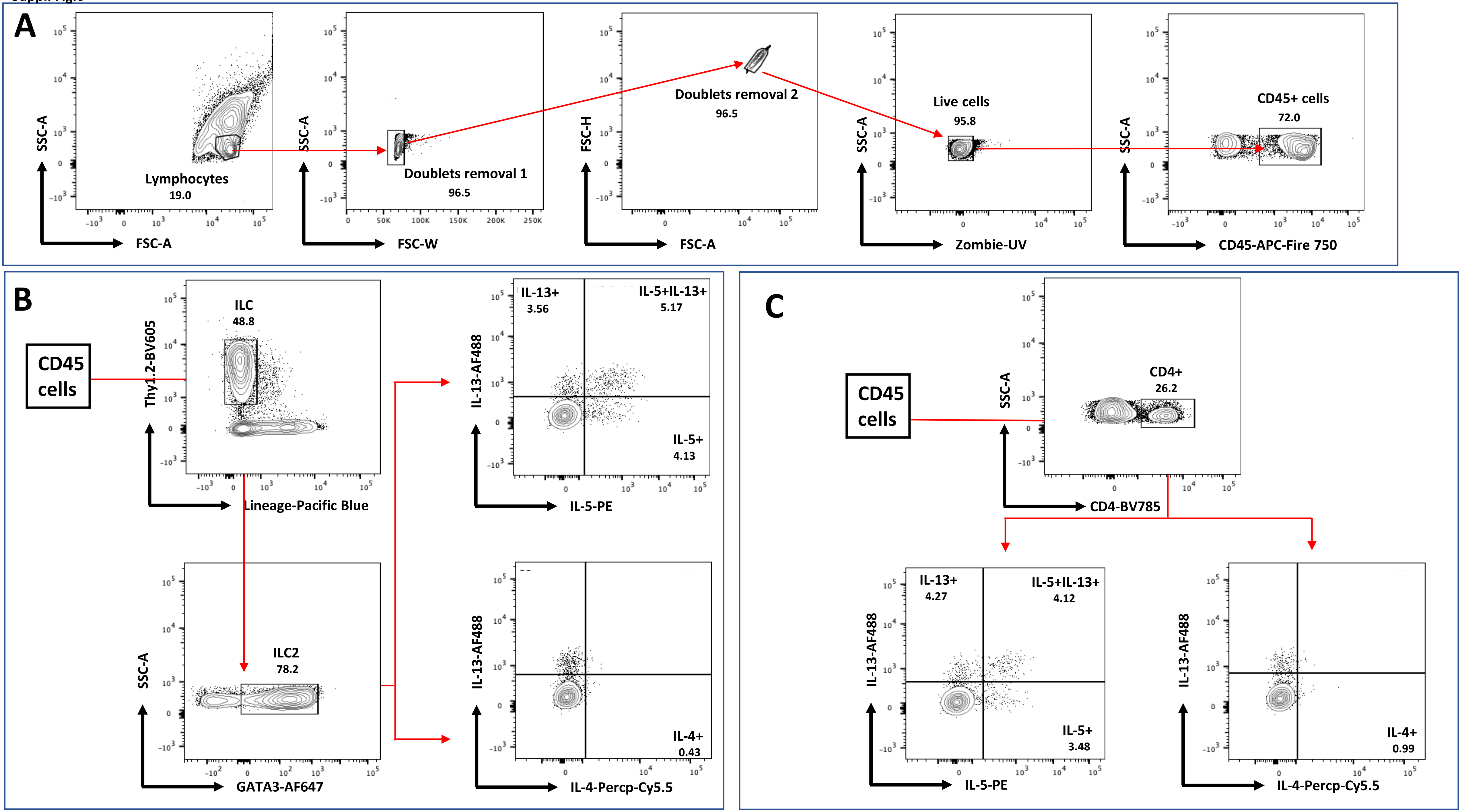

A
Lymphocytes
19.0
SSC-A
FSC-A
Doublets removal 1
96.5
FSC-W
Doublets removal 2
96.5
FSC-H
Live cells
95.8
Zombie-UV
CD45+ cells
72.0
CD45-APC-Fire 750
B
CD45 cells
ILC
48.8
Thy1.2-BV605
Lineage-Pacific Blue
ILC2
78.2
GATA3-AF647
IL-13+
3.56
IL-5+IL-13+
5.17
IL-5+
4.13
IL-13-AF488
IL-5-PE
IL-4+
0.43
IL-4-Percp-Cy5.5
C
CD45 cells
CD4+
26.2
CD4-BV785
IL-13+
4.27
IL-5+IL-13+
4.12
IL-5+
3.48
IL-4+
0.99

Suppl. Fig.7

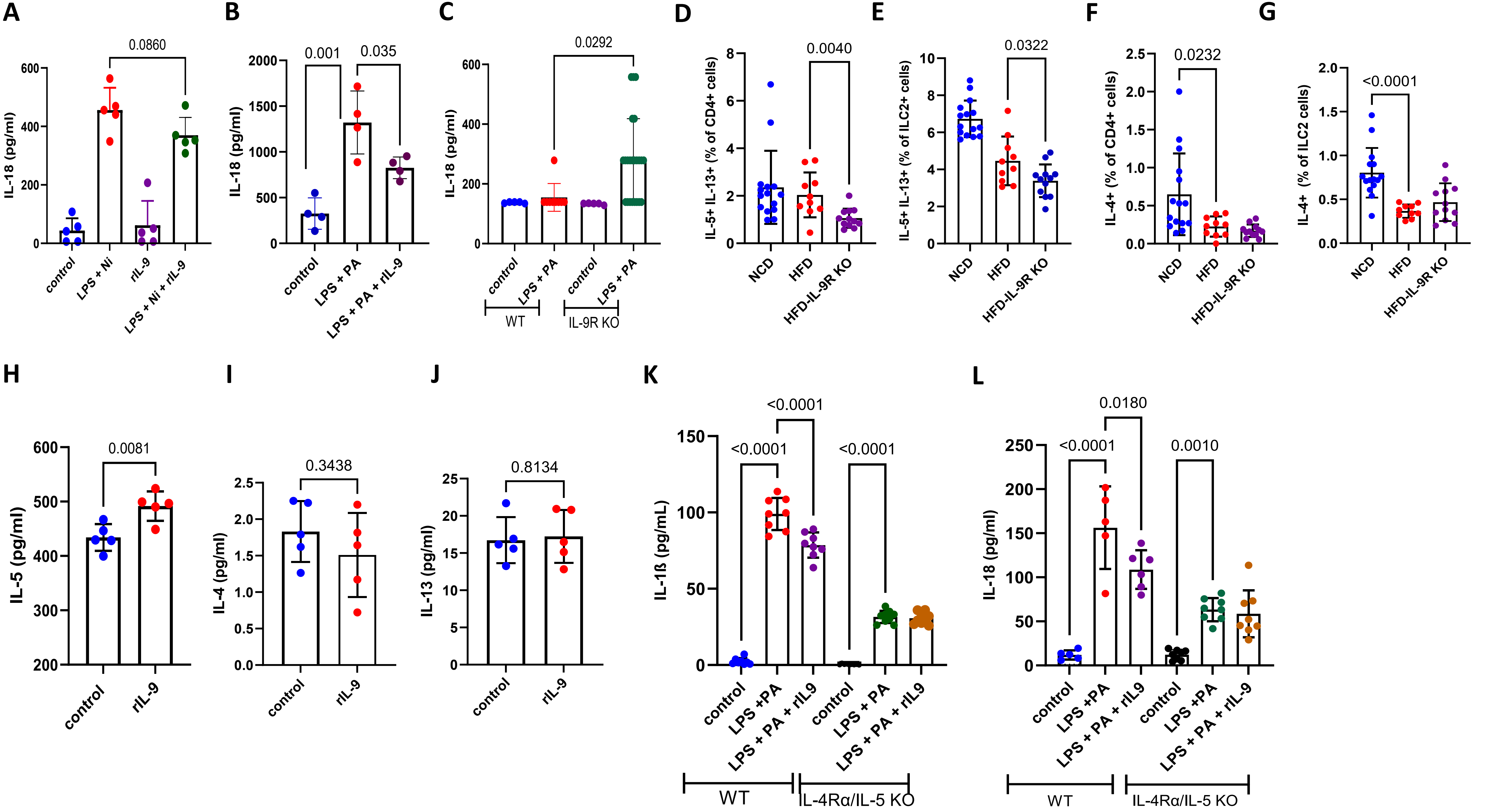
A
IL-18 (pg/ml)
0.0860
control
LPS + Ni
rIL-9
LPS + Ni + rIL-9
B
IL-18 (pg/ml)
0.001
0.035
control
LPS + PA
LPS + PA + rIL-9
C
IL-18 (pg/ml)
0.0292
control
LPS + PA
control
LPS + PA
WT
IL-9R KO
D
IL-5+ IL-13+ (% of CD4+ cells)
0.0040
NCD
HFD
HFD-IL-9R KO
E
IL-5+ IL-13+ (% of ILC2+ cells)
0.0322
NCD
HFD
HFD-IL-9R KO
F
IL-4+ (% of CD4+ cells)
0.0232
NCD
HFD
HFD-IL-9R KO
G
IL-4+ (% of ILC2 cells)
<0.0001
NCD
HFD
HFD-IL-9R KO
H
IL-5 (pg/ml)
0.0081
control
rIL-9
I
IL-4 (pg/ml)
0.3438
control
rIL-9
J
IL-13 (pg/ml)
0.8134
control
rIL-9
K
IL-1ß (pg/mL)
<0.0001
<0.0001
<0.0001
control
LPS +PA
LPS + PA + rIL9
control
LPS + PA
LPS + PA + rIL9
WT
IL-4Rα/IL-5 KO
L
IL-18 (pg/ml)
<0.0001
0.0180
0.0010
control
LPS +PA
LPS + PA + rIL9
control
LPS +PA
LPS + PA + rIL-9
WT
IL-4Rα/IL-5 KO

Suppl. Fig.8

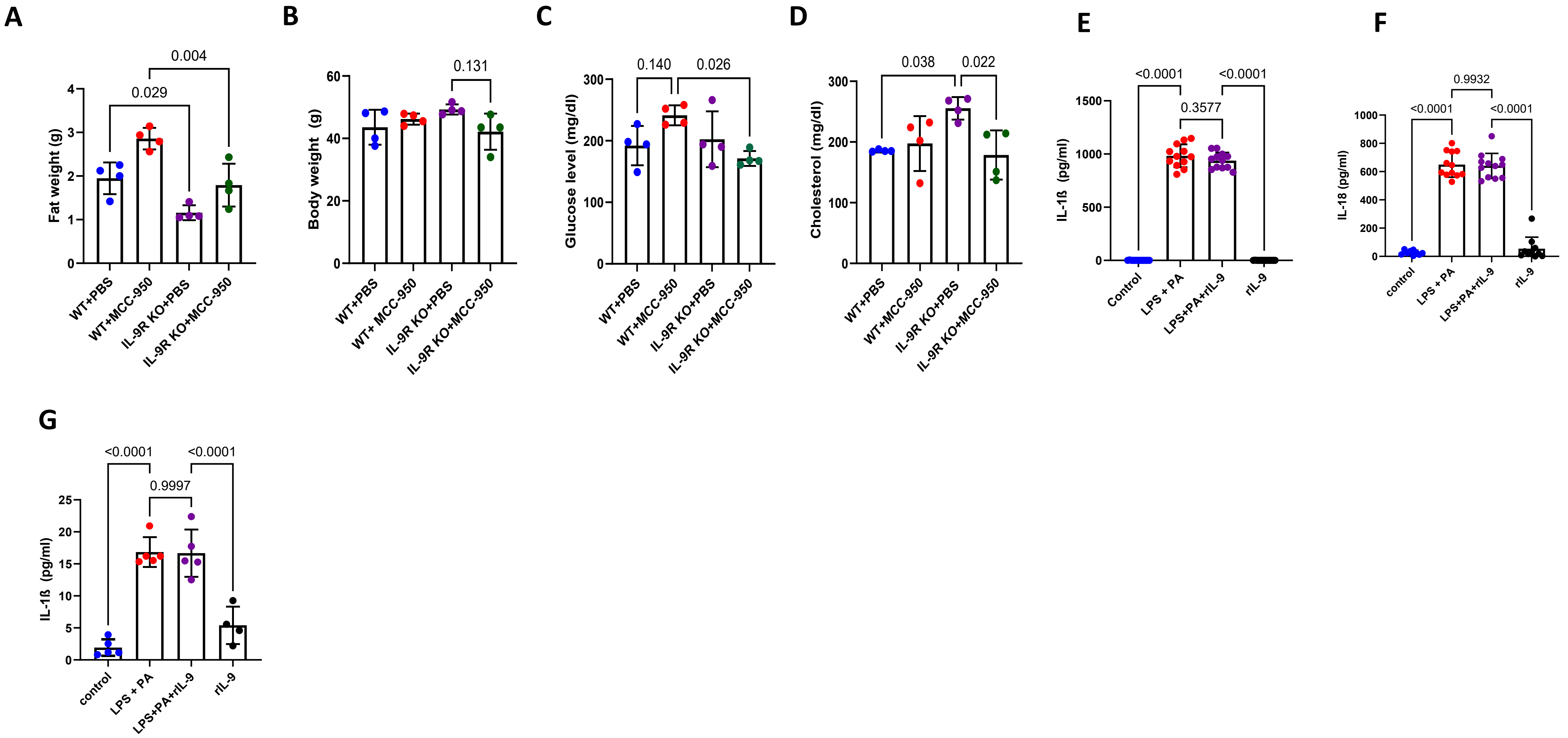
A
Fat weight (g)
0.029
0.004
WT+PBS
WT+MCC-950
IL-9R KO+PBS
IL-9R KO+MCC-950
B
Body weight (g)
0.131
WT+PBS
WT+ MCC-950
IL-9R KO+PBS
IL-9R KO+MCC-950
C
Glucose level (mg/dl)
0.140
0.026
WT+PBS
WT+MCC-950
IL-9R KO+PBS
IL-9R KO+MCC-950
D
Cholesterol (mg/dl)
0.038
0.022
WT+PBS
WT+MCC-950
IL-9R KO+PBS
IL-9R KO+MCC-950
E
IL-1ß (pg/ml)
<0.0001
0.3577
<0.0001
Control
LPS + PA
LPS+PA+rIL-9
rIL-9
F
IL-18 (pg/ml)
<0.0001
0.9932
<0.0001
control
LPS + PA
LPS+PA+rIL-9
rIL-9
G
IL-1ß (pg/ml)
<0.0001
0.9997
<0.0001
control
LPS + PA
LPS+PA+rIL-9
rIL-9

Suppl. Fig.9

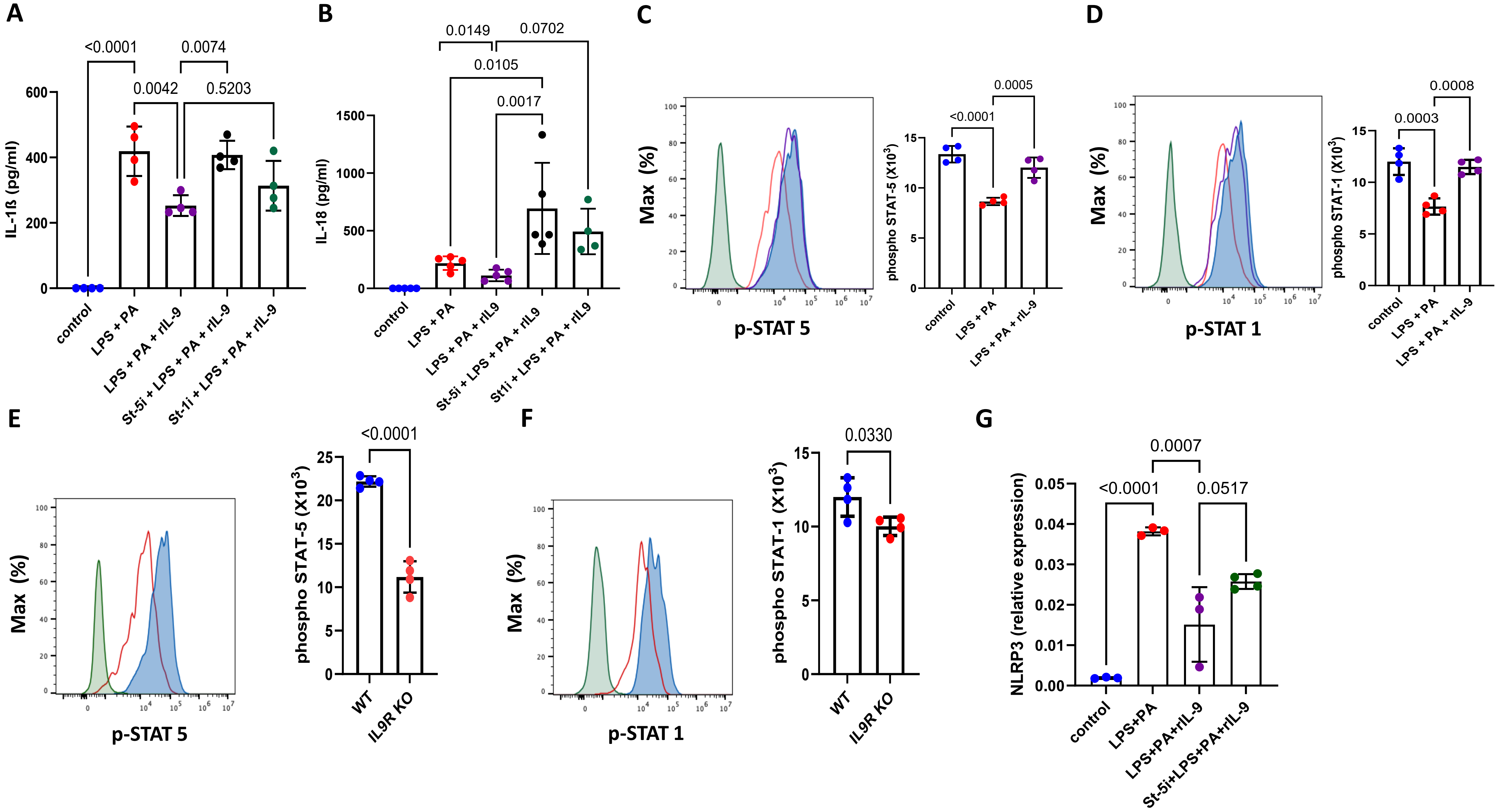
A
<0.0001
0.0074
0.0042
0.5203
IL-1ß (pg/ml)
600
400
200
0
control
LPS + PA
LPS + PA + rIL-9
St-5i + LPS + PA + rIL-9
St-1i + LPS + PA + rIL-9
B
0.0149
0.0702
0.0105
0.0017
IL-18 (pg/ml)
1500
1000
500
0
control
LPS + PA
LPS + PA + rIL9
St-5i + LPS + PA + rIL9
St1i + LPS + PA + rIL9
C
Max (%)
p-STAT 5
phospho STAT-5 (X10³)
<0.0001
0.0005
control
LPS + PA
LPS + PA + rIL-9
D
Max (%)
p-STAT 1
phospho STAT-1 (X10³)
0.0003
0.0008
control
LPS + PA
LPS + PA + rIL-9
E
Max (%)
p-STAT 5
<0.0001
phospho STAT-5 (X10³)
WT
IL9R KO
F
Max (%)
p-STAT 1
0.0330
phospho STAT-1 (X10³)
WT
IL9R KO
G
0.0007
<0.0001
0.0517
NLRP3 (relative expression)
control
LPS+PA
LPS+PA+rIL-9
St-5i+LPS+PA+rIL-9

Suppl.Table 1: Clinical and biochemical characteristics of the study subjects

Parameters	Uninfected		Infected		p value for trend
	Non-DM (N=38)	DM (N=38)	Non-DM (N=47)	DM (N=36)	
Age (years)	36.53 ± 12.37	46.00 ± 12.80*	43.91 ± 12.80*	48.80 ± 13.20*	**<0.001**
Gender (F/M)	17/21	16/22	21/26	13/23	0.858
BMI (kg/m^2)	26.73 ± 5.35	25.66 ± 5.04	24.39 ± 4.71	24.17 ± 2.91	0.055
Waist circumference (cm)	86.21 ± 11.35	84.76 ± 11.26	82.58 ± 9.46	84.38 ± 7.83	0.426
Body fat (%)	28.17 ± 13.47	27.84 ± 12.62	24.89 ± 12.87	23.18 ± 10.4	0.249
Muscle mass (%)	33.50 ± 8.411	32.68 ± 7.95	34.27 ± 8.51	35.70 ± 6.46	0.411
Systolic blood pressure (mm Hg)	118.08 ± 21.95	119.66 ± 6.27	123.35 ± 22.56	116.47 ± 17.11	0.434
Diastolic blood pressure (mm Hg)	72.32 ± 12.93	78.68 ± 12.02	73.13 ± 11.40	75.17 ± 8.90	0.071
Fasting blood glucose (mg/dl)	103.44 ± 17.69	116.95 ± 59.09	104.60 ± 18.76	104.83 ± 18.21	0.245
HbA1c (mmol/mol)	30.41 ± 9.65	63.82 ± 16.55*	34.56 ± 6.49	59.34 ±14.80*#	**<0.001**
C-reactive protein (pg/ml)	4.77 ± 3.87	15.23 ± 22.58	9.75 ± 17.85	20.90 ± 27.58	**0.006**
Total Cholesterol (mg/dl)	125.19 ± 43.34	147.08 ± 46.12	133.40 ± 37.79	128.11 ± 39.52	0.109
HDL Cholesterol (mg/dl)	57.57 ± 35.45	55.29 ± 21.70	64.70 ± 24.52	56.46 ± 32.65	0.413
LDL Cholesterol (mg/dl)	97.87 ± 51.38	109.97 ± 48.51	117.24 ± 54.47	115.11 ± 42.88	0.322
Triglycerides (mg/dl)	43.82 ± 36.70	68.64 ± 54.47*	35.21 ± 18.88	51.47 ± 31.03*	**<0.001**
Onchocera volvulus	-	-	21/40	19/40	0.516
Mansonella perstans	-	-	34/45	11/45	**<0.001**
Loa loa	-	-	28/39	11/39	**0.006**

Data are represented as mean ± SD or proportions. p for trend < 0.05 highlighted in bold.*p< 0.05 compared to uninfected-non-DM; # p< 0.05 compared to uninfected DM. One way ANOVA with Tukey honestly significant difference [HSD] was used for continuous variables and chi square test was performed for categorical variables.

Suppl.Table 2 : Spearman correlation analysis of IL-9 levels with clinical parameters

	**Body fat**	**Muscle mass**	**Insulin**	**CRP**
**Correlation co-efficient (r)**	-0.159	0.180	0.154	-0.181
**P value**	**0.046**	**0.024**	**0.052**	**0.027**
