# Supplementary material for: The role of the IL-9‒NLRP3 axis in insulin resistance and adipose tissue inflammation during diet-induced obesity
Source: Cell Mol Immunol. 2025 Sep 18;22(11):1478–90. doi: 10.1038/s41423-025-01340-4 (PMC12575696; doi:10.1038/s41423-025-01340-4)
Supplement: Supplementary file 2 — Supplementary figures and tables legends and caption [file 41423_2025_1340_MOESM2_ESM.docx]

**Suppl. Fig. 1. rIL-9 treatment does not improve glucose intolerance induced by diet-induced obesity.** (A) Glucose tolerance test (GTT) of HFD-fed mice treated i.p. with 2 µg/kg of recombinant IL-9 thrice weekly; (B) area under the curve (AUC) of GTT. Frequencies of IL-9+ (C) ILC2s (D) B cells and (E) macrophages in the SVF of NCD-fed and HFD-fed mice; (F) MFI of IL-9R expression in the immune cells of adipose tissue of NCD-fed mice; MFI of IL-9R expression in (G) CD3+ T cells, (H) eosinophils, (I) dendritic cells, (J) NK cells, and (K) CD4+ T cells of NCD-fed and HFD-fed mice. All the experiments were repeated twice; representative data are shown for A, and pooled data are shown for the other experiments. Two-way ANOVA was performed for (A), and for the remaining data, an unpaired t test was performed.

**Suppl. Fig. 2. IL-9R deficiency leads to a worsened metabolic status during HFD consumption.** (A) Representative H&E staining of visceral adipose tissue from NCD (normal control diet), HFD (high-fat diet) and HFD-IL-9R KO mice; (B) quantification of adipocyte sizes as area per adipocyte in μm (scale bar = 100 μm); (C) western blots of pAkt and total Akt in the visceral adipose tissue of HFD and HFD-IL-9R KO mice in response to insulin injection (1 U/kg body weight) 15 minutes before tissue isolation. (D) Adiponectin levels in primary white adipose tissue cultures of WT mice pretreated with rIL-9 for 24 hours and then with LPS and palmitic acid (PA) for 18–24 hours; (E) TNF levels in primary white adipocytes from WT and IL-9R KO mice treated with LPS and PA for 24 hours; (F) total cholesterol levels in the plasma of NCD, HFD and HFD-IL-9 RKO mice. (G) ITT and (H) AUC for ITT of WT and IL-9R-KO mice fed a NCD for 12 weeks. Immune cell composition: (I) Total immune cells and (J) eosinophils of WT and IL-9R KO mice fed a NCD for 12 weeks. All experiments were repeated twice; representative data are shown. Two-way ANOVA was performed for (G), and either one-way ANOVA or an unpaired t test was performed for the remaining data.

**Suppl. Fig. 3. Higher weight gain despite lower food intake in IL-9R KO mice.** (A) Weight gain; (B) food consumption during the course of long-term cold exposure; (C) amount of fat (g), free water (g) and muscle mass (g) analyzed at the end (day 10) of long-term cold exposure using a Bruker Minispec LF50H. All experiments were repeated twice, and representative data are shown. For A and C, two-way ANOVA was performed, and for the remaining data, two-tailed unpaired t tests were performed.

**Suppl. Fig. 4. Example flow cytometry gating strategy.** Representative flow cytometry gating strategy for Fig. 5B-E for the enumeration of (A) ILC2s; (B) eosinophils; (C) RELMα+ and CD11c+ macrophages from live CD45+ cells of SVF in visceral adipose tissue.

**Suppl. Fig. 5: Flow cytometric gating of eosinophils and macrophages in adipose tissue**: Example flow cytometry gating strategy for enumeration of eosinophils, total macrophages, CD11c+ macrophages and RELMα+ macrophages from live-CD45+ cells of SVF in visceral adipose tissue.

**Suppl. Fig. 6: Flow cytometric gating of ILC2s and type 2 cytokine-positive ILC2s and CD4+ T cells in adipose tissue:** (A) Gating strategy for CD45+ cells from SVF; (B) from CD45+ cells, gating strategy for ILC2s and ILC2-positive type 2 cytokines; (C) from CD45+ cells, gating strategy for CD4+ T cells and CD4+ T cells positive for type 2 cytokines.

**Suppl. Fig. 7. IL-9 signaling coordinates with other type 2 cytokines.** Bone marrow-derived macrophages (BMDMs) were pretreated with recombinant IL-9 for 24 hours and then treated with (A) LPS for 3.5 hours and nigericin for 1 hour; (B) LPS for 3 hours and palmitic acid for 18 hours, and the IL-18 levels in the supernatants were measured via ELISA. (C) Stromal vascular fraction from wild-type and IL-9R KO mice were treated with LPS for 3 hours and palmitic acid for 18 hours. Frequencies of IL5+IL-13+ (D) CD4+ T cells and (E) ILC2s, IL4+ (F) CD4+ T cells and (G) ILC2s; SVF from WT adipose tissue was treated with rIL-9 for 24 hours, and (H) IL-5, (I) IL-4 and (J) IL-13 secretion was measured by ELISA. BMDMs and SVF from WT and IL-4Rα/IL-5 KO mice were generated, pretreated with rIL-9 for 24 hours and then stimulated with LPS for 3 hours and PA for 18 hours, and the levels of (K) IL-1β in the SVF and (L) IL-18 in the BMDMs were measured by ELISA. All experiments were repeated twice; for D-G, pooled data were used, and for the remaining data, representative data are shown. One-way ANOVA was performed.

**Suppl.** **Fig. 8. Effect of NLRP3 inhibition on metabolic parameters.** (A) Visceral adipose tissue weight, (B) body weight, (C) plasma glucose and (D) cholesterol levels in HFD-WT and HFD-fed IL-9R KO mice treated with PBS or MCC-950. Bone marrow-derived macrophages were generated from Rag2IL-2Rγ KO mice, pretreated with rIL-9 for 24 hours and then treated with LPS for 3 hours and palmitic acid for 18 hours. The levels of (E) IL-1β and (F) IL-18 in BMDMs were measured by ELISA (G) SVF from Rag2IL-2Rγ KO mice were treated as described above, and the IL-1β levels were measured by ELISA. One-way ANOVA was performed. All experiments were repeated twice; representative data are shown. One-way ANOVA was performed.

**Suppl. Fig. 9. IL-9 acts via STAT-5 to inhibit NLRP3.** BMDMs were treated with inhibitors of STAT-1 and STAT-5 and rIL-9, LPS and palmitic acid (PA) as described in Fig. 6B, and (A) IL-1β and (B) IL-18 levels were measured by ELISA. Expression of (C) phospho-STAT-5 and (D) phospho-STAT-1 in wild-type (WT) BMDMs treated for 15 minutes and 30 minutes with rIL-9 respectively. MFI of (E) phospho-STAT-5 and (F) phospho-STAT-1 in WT and IL-9R KO BMDMs; (G) mRNA expression of NLRP3 in BMDMs treated with inhibitors of STAT-5 and rIL-9, LPS and palmitic acid (PA), as mentioned in A. Representative data are shown. One-way ANOVA was performed.

**Suppl. Table 1. Clinical and biochemical characteristics of the study subjects.** Data are represented as mean ± SD or proportions. p < 0.05 is highlighted in bold. One-way ANOVA with Tukey’s honestly significant difference (HSD) test was used for continuous variables, and the chi-square test was performed for categorical variables**.**

**Suppl. Table 2. Spearman correlation analysis of serum IL-9 levels with clinical parameters.**
